# Mycosporine-Like Amino Acids: Relevant Secondary Metabolites. Chemical and Ecological Aspects

**DOI:** 10.3390/md9030387

**Published:** 2011-03-21

**Authors:** Jose I. Carreto, Mario O. Carignan

**Affiliations:** National Institute for Fisheries Research and Development (INIDEP), Paseo Victoria Ocampo Street No. 1, North Pier, B7602HSA, Mar del Plata, Argentina; E-Mail: marioc@inidep.edu.ar

**Keywords:** mycosporine-like amino acids, physicochemical properties, isolation, distribution, metabolism

## Abstract

Taxonomically diverse marine, freshwater and terrestrial organisms have evolved the capacity to synthesize, accumulate and metabolize a variety of UV-absorbing substances called mycosporine-like amino acids (MAAs) as part of an overall strategy to diminish the direct and indirect damaging effects of environmental ultraviolet radiation (UVR). Whereas the enzymatic machinery to synthesize MAAs was probably inherited from cyanobacteria ancestors via the endosymbionts hypothesis, metazoans lack this biochemical pathway, but can acquire and metabolize these compounds by trophic transference, symbiotic or bacterial association. In this review we describe the structure and physicochemical properties of MAAs, including the recently discovered compounds and the modern methods used for their isolation and identification, updating previous reviews. On this basis, we review the metabolism and distribution of this unique class of metabolites among marine organism.

## 1. Introduction

Depletion of the stratospheric ozone layer, which has caused an increase in the UVB flux to the earth’s surface in recent years [1,2], could result in increased levels of UV-induced damage for most living organism [3] producing a great impact on the photosynthetic carbon fixation by plants and consequently, on the global climate change [4,5]. Recently, several studies highlight the synergistic effect of increased temperature at seasonally high levels of solar radiation, showing that a complex set of interactions between these environmental factors can lead to differential responses to a stress [6,7]. Oceanic tropical regions, in particular, experienced high ultraviolet radiation flux due to the thinness of the ozone layer at equatorial latitudes, and the high transparency of oligotrophic waters characteristic of this region [8,9]. Intertidal and epipelagic marine organisms are exposed to the highest levels of ultraviolet radiation, but surface bloom forming phytoplankton species, notably dinoflagellates, also experience a high photon flux of UV radiation [10–14]. At the beginning of the evolution of life on Earth, UVB flux rates clearly exceeded the present values [15] indicating that several protection strategies such as avoidance, screening, photochemical quenching and repair [16] have evolved to counteract the negative effects of UVR.

One of the adaptations whereby marine organisms can prevent UVR-induced damaged is the synthesis of UV-absorbing/screening substances such as mycosporine-like amino acids and scytonemin [17–22]. Considerable interest has been centered on mycosporine-like amino acids (MAAs) because experimental evidence indicated that in marine organism the major functions of MAAs are to act as photo-protective UV filters [23–26] and/or to act as antioxidants [27–29]. For example, the dinoflagellate *Gyrodinium dorsum* is able to tolerate at least twice as high doses of UVB radiation before its motility is inhibited when it has been induced to synthesize MAAs by moderate UVA radiation beforehand [13,26]. *In vitro* studies of various MAAs have also given support to this function by confirming the high photostability and the release of heat to the medium as the main relaxation pathway of the photo-excited molecules [30–32]. In addition, oxocarbonyl-MAAs such as mycosporine-glycine [27,28,33] and mycosporine-taurine [34], have antioxidant activity capable of protecting against the cellular damage that high levels of reactive oxygen species (ROS) induce in organisms under different stresses. Recently, Oyamada *et al.* [35] showed that MAAs, in particular mycosporine-glycine, have a protective effect on human cells against UV light. Further, they found a promotion effect of MAAs on the proliferation of human skin fibroblast cells. Antioxidant activity was also found in some imino mycosporines [29,34,36,37]. Therefore, MAAs, have potential applications in cosmetics and toiletries [17,35,38,39] as UV protectors and activators of cell proliferation. In addition to their photo-protective function against UVR, several other hypothesis about the role of MAAs in biological systems have been formulated, although some of them [40,41] are controversial [42] or unsupported [43,44]: (a) they may contribute to osmotic regulation [40,41]; (b) they may act as regulatory metabolites of sporulation and germination in fungi [45], and reproduction in marine invertebrates [46,47]; (c) they may act as transducers of UV wavelengths to wavelengths utilizable for photosynthesis [43,44]; (d) they may act as “host factors,” that induce release of photosynthate from the endosymbiotic algae [48]; (e) they may play a role under desiccation or thermal stress in certain organisms [41,49]; (f) they can also act as an intracellular nitrogen reservoir [50]. Recently Kicklighter *et al*. [51] showed that pyrimidines and mycosporine-like amino acids function as alarm cues in the defensive secretions of the sea hare *Aplysia californica*. The discovery that MAAs can be chemical signals raises an entirely new direction for exploring the potential functions and evolution of MAAs [52].

Studies on UV absorbing compounds in marine organisms were published as early as 1938 (Kalle, 1938 as referenced in [53]). A survey of UV absorptions in 39 species of green, brown and red algae by Tsujino and Saito [54] showed that many species of red algae contain compounds with sharp absorption in the UV region. Bon *et al.* [55] demonstrated the existence in lenses of fish, amphibians and cephalopods of components characterized by their strong absorption in the region 320–360 nm, while Shibata [56] reported finding of UV absorbing substances in water extracts from several species of corals and a cyanobacterium (likely *Trichodesmium*) from the Great Barrier Reef. Shibata speculated that these compounds (named S-320) must have a biological function such as UV-photoprotection. Soon thereafter, UV-absorbing compounds were also found in other species of red macroalgae [53,57] corals [58–61] and red tide dinoflagellates [62–66]. However, the chemical nature of these UV-absorbing substances was not known until the pioneer work of the Hirata’s group. Ito and Irata [67] first isolate and characterize mycosporine-glycine from the tropical zoanthid *Palythoa tuberculosa*, a compound with the same basic chemical structure of fungal metabolites (grouped as mycosporines) previously isolated from mycelia of sporulating fungi [68]. Since then, several closely related compounds imino-mycosporines (grouped as mycosporine-like amino acids, MAAs) have been isolated and characterized from several plants and marine animals. Contemporaneous reports indicate that taxonomically diverse marine and terrestrial organisms [17–19,22,69,70] have evolved the capacity to synthesize, accumulate and metabolize a variety of mycosporine-like amino acids (MAAs). Whereas the enzymatic machinery to synthesize MAAs was probably inherited from cyanobacteria ancestors via the endosymbionts hypothesis [71–73], metazoans lack this biochemical pathway, but can acquire and metabolize these compounds by trophic transference, symbiotic or bacterial association [17–19,22,69,70,72,74]. There are now more than 20 well characterized MAAs with maximum absorption ranging from 309 to 362 nm. In addition, several partially characterized or unknown MAAs, have been recently detected as a consequence of the increased number of studied organisms and the development of more efficient high-resolution reverse-phase liquid chromatography and mass spectrometry (HPLC-MS) techniques [11,75–79].

In this review we describe the structure and physicochemical properties of MAAs, including the recently discovered compounds and the modern methods used for their isolation and identification updating the previous reviews [17–20,22–25,80] and the database on MAAs described by Sinha *et al.* [70]. Finally we review the metabolism and distribution of this unique class of metabolites among marine organisms.

## 2. Molecular Structures and Properties

### 2.1. Structures

**Figure 1.**
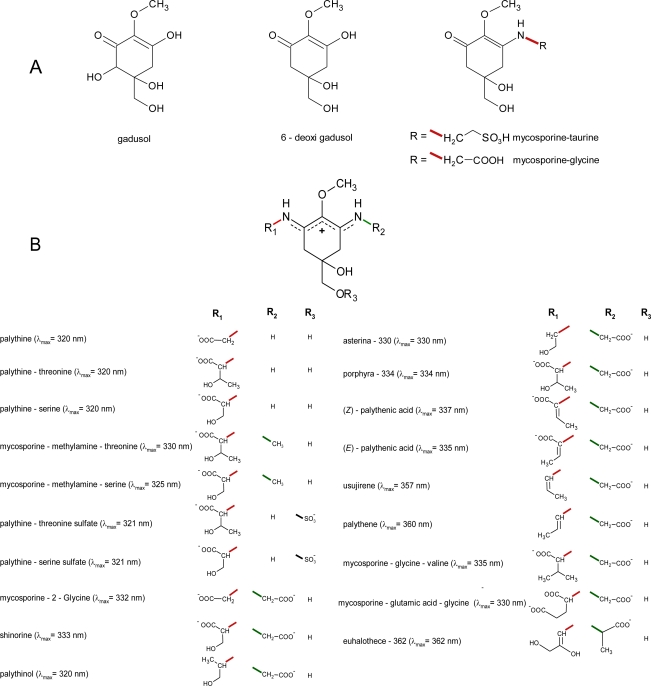
Structures of (**A**) gadusol, 6-deoxy gadusol and oxo-mycosporines; (**B**) imino-mycosporines.

In contrast to fungal metabolites [81,82], and with only two exceptions, MAAs from marine organism are imine derivatives of mycosporines which contain an amino-cyclohexenimine ring linked to an amino acid, amino alcohol or amino group (Figure 1), having absorption maxima between 320 and 360 nm [17].

Mycosporine-glycine and mycosporine-taurine are the only known aminocyclohexenones from marine sources. As fungal mycosporines [17,76,78,82,83], these compounds can be considered to be Schiff bases (enamino ketones) which posses a common ciclohexenone ring system linked with an amino acid (oxocarbonyl-MAAs), having absorption maxima at 310 nm (Figure 1A). Each MAA generally contain a glycine moiety on the C3 of the cyclohexenimine ring and a second amino acid (porphyra-334, shinorine, mycosporine-2-glycine, mycosporine-glycine-glutamic acid) amino alcohol (palythinol; asterina-330) or an enaminone system (palythene, usujirene) linked to the C1 (Figure 1B). However, in some corals glycine has been replaced by methyl amine (mycosporine-methyl-amine-serine, mycosporine-methylamine-threonine [84] or to an amine group (palythine-serine, and palythine-threonine) [79,84]. Another exception is the rare and apparently unique MAA common to several sea anemones mycosporine-taurine, a compound that has been found in the highest concentration in all the studied species [85–87].

Some MAAs isolated from corals, also contain sulfate esters [88]. Recently, a rare novel MAA, containing the amino acid alanine (2-(e)-2,3-dihydroxipro-1-enylimino-mycosporine-alanine) was isolated from the unicellular cyanobacterium *Euhalothece* sp. [77]. Another novel MAA tentatively identified as dehydroxyl-usujirene was isolated from the cyanobacteria *Synechocystis* sp. [89]. Recently, Yoshiki *et al.* [29] showed the production of a new compound from porphyra-334 by heat treatment. This compound, having λ_max_ at 226 nm and [M + H]^+^ at *m/z* 329.1394, was structurally characterized by 4,5-double bond, or 5,6-double bond with a dynamic equilibrium between them. However, the structure elucidation of many of these compounds has been not totally achieved by chemical degradation; spectroscopic methods (IR, mass spectrometry, ^1^H and ^13^C NMR; *etc.*) and X-ray analysis (see Table 1). One exception is the recently determination of the total stereostructure of porphyra-334 [90]. Although the structure of porphyra-334 was previously analyzed by Hirata and co-workers [91,92], the authors noted that their NMR assignments for C-4/C-14 and C-5/C-7 were ambiguous and might as well be reversed.

Recently porphyra-334 was extensively analyzed using ^1^H and ^13^C NMR analysis as well as to density functional theory (DFT) calculations [90]. In this study, all NMR signals of porphyra-334 including the resonances of the prochiral proton pairs could be assigned by 500 MHz standard COSY, HMQC and HMBC experiments, as well as by one-dimensional (DPFGSE-NOE) and two-dimensional (NOESY) NOE experiments. Diffusion measurements (DOSY) confirm that porphyra-334 is monomeric in D_2_O solution. DFT calculations yield ^13^C-NMR chemical shifts which are in good agreement for species with the imine N-protonated form of porphyra-334. An exceptionally high proton affinity of 265.7 kcal/mol was calculated for porphyra-334, indicating that this compound it is a powerful “proton sponge” of comparable strength as synthetic systems studied so far [90]. The absolute configuration at the ring stereo center was predicted to be S [90] which is supported by the similarities with the absolute S configuration found in mycosporine-glycine in an elaborated laboratory synthesis [93]. This results indicated that porphyra-334 is biochemically derived from 3-dehydroquinic acid via mycosporine-glycine and question the results recently published by Torres *et al*. [94] claiming that the configuration at the imino moiety of porphyra-334 is (*Z*), rather than (*E*) as established by Klisch *et al.* [90].

**Figure 2.**
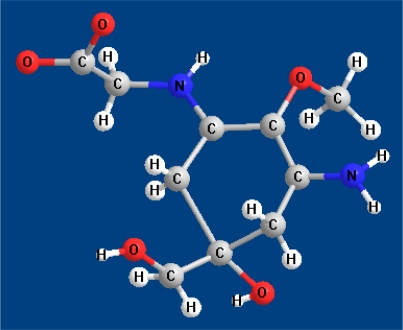
A perspective view of the palythine molecule according to Furusaky *et al.* [95].

**Figure 3.**
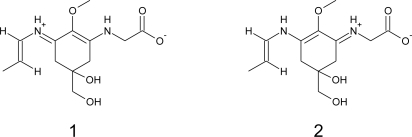
The two canonical forms for the palythene resonant hybrid [96].

However, only the crystal and molecular structure of palythine [95] and palythene [96] were unambiguously determined by X-ray analysis, including its absolute configuration (Figure 2). The structure of both compounds was defined as an inner salt and the features of delocalization of the positive and negative charge were elucidated. For example, in palythene, the positive and negative charges in the inner salt are favourable to exist in the near position each other, indicating that in the representation of this compound as a resonance hybrid, the canonical form 1 contributes more effectively than the form 2 (Figure 3). In the crystalline state (as trihydrate) palythine also exists as a zwitterion which is stabilized by resonance between two nearly-equivalent structures. In addition, the X-ray analysis showed that palythine and water molecules are connected by twelve types of hydrogen bonds, forming a three-dimensional hydrogen-bonded structure [95].

In addition to these compounds, several forms of atypical MAAs have been isolated from *Alexandrium* species [11,109–111]. One of them, originally called M-333 was tentatively identified as a mono-methyl ester of shinorine [11]. Recently their structure was confirmed (to be published). Other type of compounds, contain 2 condensed MAAs: M-320 (shinorine and mycosporine-glycine), M-335/360 (shinorine and palythene) or 3 M-328/360 (shinorine, mycosporine-glycine and palythene) [11], but the precise nature of these atypical dinoflagellate compounds is at yet unknown [11,109–111]. Additional studies revealed further novel and unidentified MAA-like compounds [11,89,111–114]. Typically in marine algae MAAs are free intracellular compounds probably concentrated around UV-sensitive organelles resulting in a high degree of packaging [115,116]. However, in some cyanobacteria, extracellular, oligosaccharide-linked MAAs (OS-MAAs) may also occur. In these compounds, the MAA chromophore is linked to oligosaccharide side chains leading to molecules that strongly interact with extracellular polysaccharides and proteins [21,117]. In algal-invertebrate symbioses MAAs were present in soluble form and not associated with any protein species whereas in asymbiotic metazoans are protein-associated and occur especially in the epidermis [19], but also in the ocular tissues of many shallow water fishes [19,118] and some cephalopod mollusc such as *Sepia officinalis* [119]. Asterina-330 and gadusol exist in association with soluble proteins in fish lenses. The highly unstable major complex with absorption λ_max_ = 330 nm, when dissociated, yielded asterina-330 (λ_max_ = 330 nm) and a protein of MW 80–100 kDa (λ_max_ = 280 nm). The second, relatively “stable” minor complex of λ_max_ = 323 nm, under similar conditions, yielded gadusol (λ_max_ = 269 nm) and a protein of MW 20–30 kDa (λ_max_ = 280 nm) [17]. A recent study of diatom frustules-bound organic matter in opal-rich Southern Ocean plankton and sediments, suggested for the first time the presence of MAAs in close association with a mineral phase [120].

### 2.2. Properties of MAAs

#### 2.2.1. Spectral Characteristics

Mycosporine-like amino acids are thought to be the strongest UVA-absorbing compounds in nature [121]. Marine mycosporines which posses a common ciclohexenone ring system and a metoxy moiety at carbon 2 showed the same spectral characteristics, including the position of their absorption maximum (λ_max_ = 310 nm in H_2_O). MAAs with different molecular structure but identical ring-chromophores also had very similar spectral characteristics. Due to the lack of fine spectral absorption, the only spectral characteristics available for MAA identification were the position of the absorption maximum (λ_max_). This spectral characteristic and the value of their extinction coefficient (ɛ) are influenced by the nature of the groups linked to the amino-cyclohexenimine ring. The wavelength of the absorption maximum, range from λ_max_ = 320 nm (in H_2_O) for the non-substituted amino-MAAs (palythine, palythine-serine and palythine-threonine) to λ_max_ = 360 nm (in H_2_O) for the substituted amino-MAAs palythene. Molecular structures of palythene (*trans*-isomer) and usujirene (*cis-*isomer) and the isomeric pair *E/Z* palythenic acid, differ from those of other MAAs by the presence of a double bond conjugated with the cyclohexenimine chromophore in one of the imino substituents. The degree of resonance delocalization in a molecule can affect the energy requirements of an electronic transition and therefore affect the position of their absorption maximum and also the value of their extinction coefficient. The more efficient is the electron delocalization in a molecule, the higher its extinction coefficient. Therefore, among MAAs, palythene exhibited the higher [17] molar extinction coefficient (ɛ = 5 × 10^4^ L·mol^−1^·cm^−1^). In general the *cis*-isomers showed a hypsochromic displacement of 2–3 nm with respect to their *trans* analogues, as shown for the isomeric pairs palythene (λ_max_ = 360 nm in H_2_O) and usujirene (λ_max_ = 357 nm in H_2_O) and for the isomeric pairs *Z*-palythenic acid (λ_max_ = 337 nm in H_2_O) and *E*-palythenic acid (λ_max_ = 335 nm in H_2_O) (Figure 1B). The observed hypsochromic shift in usujirene could be due to a steric conflict between the methyl group and the nitrogen atom of the enaminone system [96], forcing the double bond of the conjugated substituent out of planarity with the cyclohexenimine chromophore. This twisting reduces the conjugation between the chromophore ring and the double bond of the substituent, thereby becomes spectrally less efficient than their *trans*-analogue. In general, *cis*-isomerization also causes a hypsochromic change in the molar extinction coefficient of conjugated compounds [122]. The extinction coefficient of usujirene has not yet been determinated although it probably does not differ significantly to that determined for palythene [123]. Wavelength absorption maxima for many specific MAAs are identical or are only 2–3 nm apart (Figure 1B) which makes it difficult to distinguish these compounds based in absorption spectra only. In addition, besides the nature of the moieties linked to the chromophore, the UV-vis spectral properties of MAAs are also affected by temperature and by the pH [124] and nature of the solvent [30,125]. For some MAAs/solvent combinations, the observed solvent-spectral changes are small, e.g., the shifts will usually be within 2–4 nm [30,125]. The effect of pH has been studied by Zhang *et al.* [124]. These authors reported that in high acidic aqueous solutions (pH = 1–3) the absorption maximum of porphyra-334 showed a hypsochromic shift to 332 nm in the pH = 3 solution and to 330 nm in the pH = 1 and pH = 2 solutions. In these conditions the extinction coefficient also decreased with the increase of acidity. As other MAAs, porphyra-334 is a zwitterion (Figure 1B) and in acidic conditions with a pH below 3.0, the protonation of the unbounded ion pair electrons of nitrogen atoms would prevent the resonance delocalization of Π-electrons in the molecule [124]. On the other hand, the absorption maximum and extinction coefficient of porphyra-334 do not change in alkaline solutions [124].

#### 2.2.2. Photo-Induced Transformations

Although many studies have focused on the presence and potential role of MAAs, there are a few reports on the photo-induced transformations of algal imino-mycosporines *in vitro* [30–32,123,126]. Some previous results showed that in aqueous solutions of shinorine, no significant change in concentration was observed after 24 h of UV irradiation [24]. Similarly, MAAs extracted from a red alga *Gracilaria cornea* were irradiated at 75 °C in distilled water and no significant change in MAA absorption was observed [127]. Studies on the excited-state properties and photostability of MAAs in aqueous solution demonstrated that the lack of fluorescence and radical production observed for porphyra-334 [30] and for the structural related MAA shinorine [31,126] and palythine [32], together with their high degree of photostability *in vitro*, support the photoprotective role assigned to these MAAs. In fact, photodecomposition quantum yields of these imino-mycosporines are very low and range between 1.2 × 10^−5^ for palythine [32] to 3.4 × 10^−4^ for shinorine [31]. Anoxic conditions seem to enhance the photostability of some MAAs since quantum yields for shinorine and porphyra-334 are reduced in *ca.* 40% on going from oxygen-to nitrogen-saturated solutions [31]. The non radiative relaxation as the main deactivation pathway of the excited states of these imino-MAAs *in vitro* has been directly quantified by photoacoustic calorimetry. The results confirm that in general more than the 90% of the excited energy is released as heat to the molecular surroundings [31,32]. The photochemistry of oxo-mycosporines has not been systematically studied yet. However Suhn *et al.* [28] observed that virtually no degradation of mycosporine-glycine takes place when irradiating methanolic solutions under anaerobic conditions. Besides, the photoinduced reactivity of the structurally-related keto-enolic compound known as gadusol has been recently assessed in aqueous solution. The results also indicate a high degree of photostability, although in this case, it depends on the acidity of the media. Thus, the photodecomposition yield for gadusol under neutral conditions is smaller in around a factor 100 than the yield at lower pH [128].

On the other hand, the role of MAAs as transducer of the absorbed UV to wavelengths utilizable for photosynthesis [43,44,129] appear to be incompatible with the very low quantum yields of fluorescence observed for aqueous solutions of shinorine and porphyra-334 [30,31,126,130]. Conde *et al.* [123] also demonstrated that irradiated aqueous solutions of usujirene, the expected more photoreactive MAAs, showed a low photodecomposition quantum yield (*Φ*_u_ = (2.86 ±0.80) × 10^−5^), which can be partially accounted for the *cis-trans* photoisomerization of usujirene to palythene (*Φ*_u→p_ = (1.71 ±0.13) ×10^−5^). However, palythene in aqueous solution showed a higher photostability than did usujirene under equivalent conditions, establishing a photostationary mixture of *cis-trans* isomers with a relative composition of palythene:usujirene = 11:1. While pure solutions of MAAs have shown a high degree of photostability, there are evidences that they are susceptible to photosensitization [31,131]. Photodegradation rate constants for palythine under polychromatic irradiation in the presence of riboflavin or rose of Bengal, and also with sea water medium, have been recently determined [132]. This study further highlights the photostability of several MAAs in both distilled and seawater in presence of photosensitizers.

Laser-flash photolysis experiments have lead to the characterization of the excited triplet state of the MAAs shinorine, porphyra-334 and palythine ([30–32]. Data for the triplet-triplet energy transfer from 1-naphthalene-methanol to porphyra-334 was used to assess the quantum yield of the MAA triplet production in N_2_-purged solutions amounting *ca.* 3% [31]. This result accounts for the low probability of triplets to generate reactive intermediates which might start further chemical reactions, in agreement with the lack of radical detection reported by Shick *et al.* [126]. The energy levels of the triplet states (ET) were estimated from the evaluation of the rate constants for the energy transfer processes with donors of variable triplet energies. Thus, ET for porphyra-334 in water solution is delimited by a value of <250 kJ·mol^−1^ whereas ET for palythine yields approximately 330 kJ·mol^−1^ [30,32]. On this basis, porphyra-334 but not palythine may be able to effectively quench the excited state of thymine with a triplet energy in DNA estimated in 270 kJ·mol^−1^ [133]. This quenching reaction by MAAs has been proposed by Misonou *et al.* [134] as a protection mechanism against UV-induced damage in the red alga *Porphyra yezoensis*, in addition to the simple filtering effect [134].

#### 2.2.3. Chemical Reactivity

Some mycosporines are remarkably susceptible to hydrolysis. For example, mycosporine-glycine was converted to ß-diketone (6-deoxygadusol) and glycine when heated in water at 80 °C for 3 h [135]. Mycosporine-glycine was rather unstable under aerobic conditions [135] and therefore should be preferably converted by standard methylation with diazomethane to the stable methyl ester [104,135]. Many MAAs, were stable in acidic media, and on treatment with HCl-methanol can be converted to their methyl esters. Hydrolysis of MAAs with alkali under several conditions yielded the amino acid or amine subunits and different derivatives [104,135]. Temperature also had marked influence on the stability of some MAAs. The most resistant one appears to be porphyra-334. At room temperature, porphyra-334 was stable in solutions with pH from 1 to 11 for 24 h, but rather instable at 80 °C [124]. Sinha *et al.* [127], found that the absorption properties of porphyra-334 and shinorine were unaffected when subjected to heat treatment at 75 °C for up to 6 h. Yoshiki *et al.* [29] showed that porphyra-334 from extracts of *P. yezoensis* (Nori) at temperatures over 100 °C produces a dehydrated compound that exhibited an extremely high antioxidant activity, comparable with that of the resveratrol, a potent natural antioxidant [136]. However, some MAAs are markedly unstable. Carreto *et al.* [75] reported that in 25% aqueous MeOH at 45 °C during 2 h, the hydrolysis of shinorine methyl ester and of the complex MAA M335/360, occurred with the concomitant increase of shinorine. In addition, the native palythene and the palythene produced by the hydrolysis of M-335/360 were partially degraded to palythine. Similar hydrolytic cleavage of an unknown MAAs, probably shinorine methyl ester, with the corresponding increase in the proportion of shinorine was observed by Tartarotti and Sommaruga [137] in freshwater phytoplankton samples. In addition, usujirene and palythene are unstable in acidic medium and yielded palythine by treatment with diluted hydrochloric acid [10,104]. A similar cleavage was observed for these MAAs after standing in a pH = 3.15 solution at ambient temperature for 24 h [70]. On the other hand, mycosporine-glycine, mycosporine-taurine and usujirene, are probably highly susceptible to oxidation by air/oxygen [27,28,135,138,139]. Dunlap and Yamamoto [27] demonstrate that mycosporine-glycine was consumed in a reaction with the hydrophilic stable free radical 2,20-azo-bis (2-amidinopropane) dihydrochloride (AAPH). In contrast, the iminomycosporine-like amino acids shinorine, porphyra-334, palythine, asterina-330, and palythinol were not oxidized [19,27]. Mechanistic studies have hypothesized that the greater susceptibility of mycosporine-glycine to oxidation, and thereby, its efficacy as an antioxidant is related to its lower redox potential and greater ability to act as a reducing agent in donating an electron to stabilize a free radical [27]. More recently, mycosporine-glycine was reported to inhibit singlet oxygen (^1^O_2_) mediated type II photosensitization in not only eosin Y-mediated red blood cell hemolysis, but also methylene blue-mediated lipid peroxidation of soybean hypocotyl microsomes [28]. The ^1^O_2_ quenching efficacy of mycosporine-glycine may conceivably be associated with the carbonyl group of the cyclohexenone chromophore and some resonance stabilization by the carbon ring double-bond structure. On the other hand, the inhibition of AAPH derived peroxyl radical-induced lipid peroxidation by mycosporine-glycine may be associated with hydrogen abstraction from the cyclohexenone ring at C-4 or C-6 with resonance stabilization provided, as proposed by Nakayama *et al.* [26] for usujirene. Nakayama *et al.* [26] indicated that usujirene has also an antioxidant effect on autoxidation of linoleic acid. The high antioxidant activity of usujirene might be explained by the fact that usujirene seems to easily donate a hydrogen atom from C-4, C-6, or C-9 methylene to a free radical of a lipid such as linoleic acid (LOO^•^), because the free radical formed in the molecule is stabilized by resonance in conjugation with double bonds of the *cis*-unsaturated chain at C-11 on the double-bonded nitrogen in series with the carbon ring double-bond structure. In a recent study, de la Coba *et al.* [37] analyzed the effect of pH on the antioxidant activities of several partially purified. Their results showed that mycosporine-glycine had the highest activity at all pH values tested. Maximum antioxidant activities were obtained at pH 8.5. At this pH mycosporine-glycine was 8 times more effective than the control L-ascorbic acid, whereas the other MAAs (asterina-330 + palythine, porphyra-334 and shinorine) showed lower antioxidant activity. The antioxidant activity decrease with pH, probably because, the anionic character of the molecules decreases due to the protonation of amine and carboxyl groups. In this case, the conjugated amino groups are less susceptible to the oxidation [37].

### 2.3. Methods for MAAs Isolation, Identification and Quantification

#### 2.3.1. MAAs Isolation

Chromatographic techniques involving gel permeation [61,91,105,135], ion exchange resins [10,11,30,61,91,97,101,105,107,135,140]; norite A [91], carbon [35,105,107] and cellulose columns [141], preparative TLC on silica gel [10,104] and semi-preparative or preparative HPLC and UV detection [11,35,84,85,88,103,142] have all been used in different combinations for the isolation and purification of MAAs and can be used to prepare reference MAAs for HPLC calibration [143].

As for other natural drugs, the fully structure elucidation of MAAs has been achieved by chemical degradation, spectroscopic techniques (UV-spectra, IR-spectra, ESI-MSn; ^1^H and ^13^C-NMR, *etc.*) and X-ray analysis. However, in many cases the MAAs was isolated in small amounts and the structure characterization was only based from the molecular mass of the isolated compound and the amino acids yielded by their alkaline hydrolysis. Obviously, the technical details for the isolation of a specific MAA will depend on its polarity and the biological source used. In addition, the selection of the biological reference material and the methods for MAAs purification also depends on the available material and the needed amount. The amount and diversity of interference substances are higher in marine animals than in algae, and in many cases more sophisticated methods are needed. In our experience, purification on Dowex 50W and carbon column followed by semi-preparative reverse-phase HPLC is the most convenient combination [11,46,79,101,107,144], and gives good results. For the methods used in MAAs identification see Table 1.

#### 2.3.2. HPLC-Approach for the Identification and Quantification of MAAs

Methods used for extraction, separation and identification of MAAs has been reviewed by Carreto *et al.* [75,143] and readers should consult these papers for detailed information about sample storage, extraction, interferences and clean-up. Papers of Volkmann and Gorbushina [78] and Karsten *et al*. [145] can be also consulted for the analysis of fungal mycosporines and for the routine analysis of MAAs in macroalgae, respectively. Ingalls *et al.* [120] found that MAAs in cleaned hydrofluorhydric acid (HF) digested diatom frustules were up to two orders of magnitude more abundant than methanol extractable MAAs. Hence the concentration and diversity of MAAs in diatoms may be much higher than previously expected [70,111,112].

##### 2.3.2.1. HPLC-Separation

**Figure 4.**
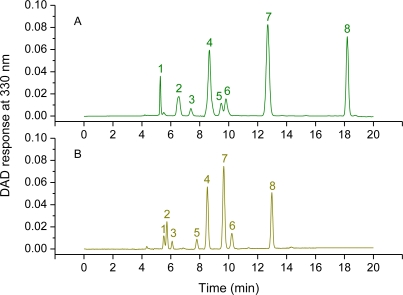
HPLC-DAD chromatograms of *Pocillopora capitata*. (**A**) HPLC conditions as in Carreto *et al.* [75] and (**B**) HPLC conditions as in Carignan *et al.* [79], reprinted with permission from Elsevier. 1: shinorine; 2: palythine-serine; 3: palythine; 4: porphyra-334; 5: mycosporine-methylamine-serine; 6: mycosporine-glycine; 7: palythine-threonine; 8: mycosporine-methylamine-threonine.

Techniques for the separation of mycosporines and MAAs of marine organisms, included those of Nakamura *et al.* [146]; Dunlap and Chalker [61] and their modifications [85,147]; Karsten *et al.* [145]; Carreto *et al.* [11,75], Ingalls *et al.* [120] and Llewellyn and Airs [111]. The classical HPLC method was based on reverse-phase low silanol-free group octadecylsilica (C_18_) columns and isocratic elution with 0.02% acetic acid as mobile phase at 15 °C [146]. Recently Volkmann and Gorbushina [78] using isocratic C_18_ (endcapped) reversed phase liquid crhomatography/mass spectrometry has successfully separated and identified eight different mycosporines of terrestrial fungi and five cyanobacterial MAAs. Dunlap and Chalker [61] first introduced the use of monomeric octylsilica (C_8_) columns. The original method was based on reverse-phase non-endcapped C_8_ column and isocratic elution with 0.1% acetic acid and 10% methanol. Under these conditions the more weakly acidic compounds were separately eluted. However, it was unable to separate mycosporine-2-glycine from porphyra-334 and mycosporine-glycine from mycosporine-taurine. Stochaj *et al.* [85] showed that the chromatographic separation of the acidic compounds could be improved if the methanol content of the mobile phase was increased up to 75%. In this condition, the highly polar compounds interacted with the weak anion exchange properties of the silanol groups to give an improved chromatographic separation of these compounds. Although these methods and their further modifications achieved good separation of several compounds [147], none of these was able to separate in a single run the strongly acidic MAAs and the more weakly acidic compounds. Separation of these mixtures was only achieved by isocratic elution with two different mobile phases [25,86,148,149]. However, resolution for some critical pairs as mycosporine-glycine and mycosporine-taurine and the MAAs-sulfated esters, was insufficient. Even the most recent modifications of these methods do not completely resolve these MAAs mixtures. Carreto *et al.* [11] showed that using acetonitrile-based eluents and polymeric double endcapped C_18_ columns, a mixture of strongly acidic and neutral MAAs can be separated in a single run. Nevertheless, this method failed in the separation of certain highly polar MAAs (shinorine from mycosporine-2-glycine and palythine-serine), characteristic of some scleractinian corals [84,88,142,147]. A high-resolution HPLC method based on reverse-phase C_18_ column and a mobile phase containing trifluoroacetic acid (TFA) and ammonium was developed by Carreto *et al.* [75]. The method is selective enough to resolve in a single run a complex mixture of over 20 MAAs, including the critical and highly polar compounds shinorine, mycosporine-2-glycine and palythine-serine, the medium polarity pair palythenic acid and shinorine methyl ester and the low polarity isomeric pair usujirene and palythene. Good precision was obtained in the separations.The relative standard deviation for retention times was below 1% and the mean relative standard deviation for integrated area estimations was below 2%. A mean column recovery of standards was 99% (±1%). The applicability of the method [75,79,87,109] was tested using extracts of microalgae cultures, natural phytoplankton populations, scleractinian corals and several species of sea anemones. The selectivity of the method towards some recently discovered MAAs makes it especially suitable for studying not only new field samples, but also for re-examination of the MAA composition of previously studied organisms [75,79]. Recently the chromatographic conditions, after small methodological changes, have been adapted for mass tandem spectrometry detection (HPLC-MS/MS). TFA enhances retention by ion pairing with the MAAs and improves peak shape by reducing silanol interactions. However, TFA has adverse effects on ESI-MS detection. Its high surface tension prevents efficient spray formation and TFA ions in the gas phase ion-pairing with the MAA’s basic groups suppressing their ionization and reducing the MS signal [150]. Because these adverse effects, the mobile phases containing 0.2% TFA-ammonium solution (pH = 3.15) [75] was replaced by a 0.2% formic acid solution [79]. The use of formic acid greatly improves the signal of the less polar MAAs, but resolution is poorer (Figure 4).

A similar approach, but using a C_8_ column (Zorbax Eclipse XDB) and a binary solvent composed by a 0.2% formic acid solution (solvent A) and MeOH (solvent B) was recently employed by Ingalls *et al.* [120]. A compromise between MAAs resolution and sensitivity can be obtained by decreasing the concentration of TFA [29] or adding small amounts of TFA to formic or propionic acid [150,151]. Another HPLC method based on anion exchange using a Luna NH_2_ column (Phenomenex; 250 mm × 4.6 mm; 5 μm particle size) and gradient elution with photodiode array (PDA) or ESI-MS detection was recently developed by Llewellyn and Airs [111]. Nevertheless, the resolution and sensitivity of these methods has not been tested for complex MAAs samples.

##### 2.3.2.2. Identification

Mycosporine-like amino acids are usually identified based on their retention time during HPLC and their characteristic UV absorption spectra obtained via diode array detection (DAD). Although DAD allows the fast acquisition of UV-VIS absorption spectra, the lack of fine spectral absorption, the influence of pH and, for some specific MAAs, wavelength absorption maxima identical or only a few nm apart (Figure 1B), makes it very difficult to distinguish MAA compounds based on absorption spectra only. In addition, a common difficulty has been the lack of commercially available standards, the preparation of which is costly and time consuming [143]. As a result, detection, identification and quantification are most usually based on co-elution with secondary standards provided by other researchers and/or comparison with published UV spectral data and HPLC retention times. Such characterization of these compounds should be treated with caution [17,148].

**Figure 5.**
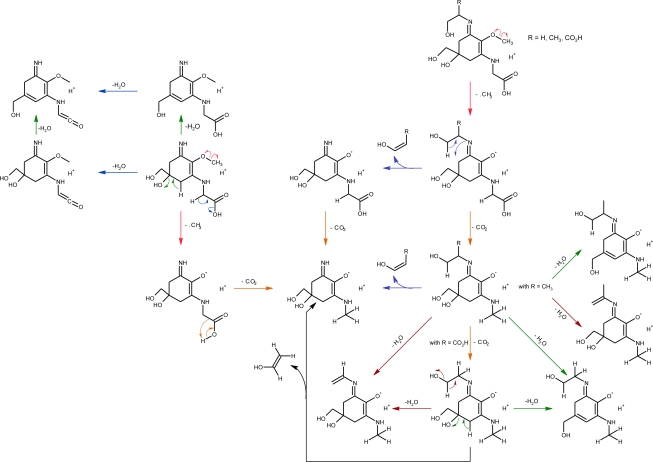
General mechanisms of fragmentation proposed for the studied MAAs in the high resolution mass spectrometry study by Cardozo *et al.* [106].

Mass spectrometry detection (HPLC/MS) using pure reference compounds, can make an invaluable contribution in the identification of MAAs because of its high sensitivity, and the availability of powerful tandem mass spectrometric techniques [29,78,79,98,106,120,148,152–155]. Atmospheric pressure ionization is the currently most used ionization technique for quantitative LC–MS methods. Two general subtypes of this technology exist: electrospray (ESI), and atmospheric pressure chemical ionization (APCI). Both are considered as “soft” ionization procedures and both produce mainly protonated or deprotonated ions without fragmentation [156]. However, using an ESI-MS approach, [152] observed that maintaining all other MS settings constant, a higher cone voltage (>40 eV) than that needed to obtain the molecular ion, produce an increased extent of MAA fragmentation. The mass spectral approach, add to LC/DAD the capability to distinguish MAAs based on their molecular weights. However, in addition to the isomeric pairs *E/Z* palythenic acid and usujirene/palythene, the protonated molecules of palythine-threonine, asterina-330, and mycosporine-methylamine-serine exhibited the same *quasi-*molecular ion (*m/z* 289). Therefore, although ESI-LC-MS is a robust technique for the analysis of MAAs, a high chromatographic resolution should be used for unambiguous identification of MAAs. ESI-MSn analysis is a more robust technique suitable not only for identification of MAAs [29,78,98,111,120,152,154] but also for structure elucidation of novel MAAs [29,79]. Moreover, the ESI-MS/MS experiments employing deuterium labeling can suggest which groups were present in the basic skeleton molecule [79,98]. For example, the hydrogen on an unsubstituted amino functionality, such as palythine, was interchangeable with deuterium atom; whereas on other MAAs containing imines, it did not occur [98]. Whitehead and Hedges [152] using a triple-quadrupole ESI-MS/MS obtained the positive-ion mass spectral fragmentation of seven (shinorine, porphyra-334, palythinol, asterina-330, palythine, palythenic acid and palythene) purified MAAs, presenting for the first time a descriptive overview of their fragmentation pattern. Results showed unusual, small radical losses of 15 mass units for five of the tested MAAs, with the exceptions of shinorine and porphyra-334 for which the initial losses were 30 and 44 mass units, respectively. As expected from structural similarities, porphyra-334 and shinorine showed similar fragmentation pattern, and also occurs for palythenic acid and palythene. Several compounds show abundant ions at *m/z* 230 and 244, which are possible the common ring system of the MAAs. Other small neutral losses of 44 (CO_2_) and 18 (H_2_O) mass units were repeatedly and commonly observed in the MAAs [143]. Whitehead and Hedges [152] suggested, based on observations from the analysis of polyamines in ion-trap mass analysis that the small radical loss of mass 15 could be due to elimination of a methyl radical. Recently, Cardozo *et al.* [106] in a high resolution mass spectrometry study (MSn) of the fragmentation of shinorine, palythine, palythinol and asterina-330, demonstrated that the elimination of mass 15 is a radical processes taking place at the methoxyl substituents of the double bound (Figure 5).

Theoretical calculations suggest that the occurrence of [(M + H) − 15]^+^ is generated by the cleavage of the ether bond through reinforcement of the C(7)–O(14) bond and the consequent weakening of C(14)–O(15) bond. Whereas the detection product ions [(M + H) − 15]^+^ and [(M + H) − 59]^+^ related to the initial elimination of the methyl radical, subsequently CO_2_ was ubiquitous in most MAAs studied. On the other hand, the fragmentation of porphyra-334 a bisubstituted MAAs containing threonine [152,154] and their structural related MAA shinorine [98,154], is unusual and showed a pathway mainly via the initial loss of CO_2_ [(M + H) − 44]^+^.

Cardozo *et al.* [154], based on experimental and theoretical computational analysis, suggested that the presence of the two acidic functions changes the driving force for the fragmentation with a neutral elimination of CO_2_ being the predominant first step with all instrument set-ups. These results are in agreement with our studies on fragmentation patterns of shinorine methyl esters; where the loss of a methyl radical by the homolytic cleavage of the O–C bond was observed to be the preferred initial fragmentation pathway. However, the spectra of the monocarboxylic MAA mycosporine-threonine clearly show the predominant initial loss of CO_2_; the radical fragmentation route occurred but only at very low intensity [79]. The resulting isolated ion (*m/z* 245), can lose H_2_O by a neutral elimination and part of the threonine chain (C_3_H_6_O) to yield the ion *m/z* 169 [79]. LC-MS/MS analyses of the coral *Pocillopora capitata* MAA extracts showed that in addition to porphyra-334 and mycosporine-threonine, the fragmentation pattern of mycosporine-methylamine-threonine was also characterized by the predominant initial elimination of CO_2_ [79]. In the case of palythine, in addition to the predominant fragmentation via the radical pathway, the neutral loss of water (18 mass units) also occurred, but only at very low intensity [98,152]. Probably, the neutral loss of water occurs from two different portions of the molecule (Figure 5). These observations suggest that the analytical methodology (such as quantification) based on protonated molecules and radical fragmentation route is not always a good choice for all MAAs.

##### 2.3.2.3. Quantification

The commonly used method for MAA detection and quantification is separation by HPLC followed by DAD identification and quantification. For this purpose, a series of known masses of pure MAA standards are injected and the resultant chromatographic peak areas are related to injected masses to yield a response factor for each MAA. The masses injected of each compound could be quantified using their specific extinction coefficients (ɛ) and the dilution factor. However, the extinction coefficient of some MAAs has not been determined [143]. In this case, the use of the extinction coefficient for the MAA which has the closest match in wavelength maxima may aid in yielding a useful concentration estimate [75].

A careful evaluation of matrix effects has to be an integral part of the validation of quantitative methods in LC-DAD. UV-absorbing substances unrelated to MAAs, were also present in extracts of marine organisms. Evaporation of the methanol extract under reduced pressure prior to HPLC injection and re-dissolution of the residue with water or water acidified mobile phase can remove water insoluble materials [11,147]. Solid phase extraction (SPE) on C-18 cartridges gave good results [84,142,147] and specifically removed substances such as pigments and lipids that not only affect sample analysis but also significantly shorten the life of the columns. However, the efficiency for individual MAAs recovery has not been quantitatively determined. Ultra-filtration is another alternative that, in addition to the water-insoluble material, removes molecules larger than the membrane molecular weight cut-off rating. Nevertheless, highly polar, low molecular weight UV-absorbing substances unrelated to MAAs were generally present in extracts of marine organisms. As these interferences were not removed by solid phase extraction or ultra-filtration, care should be taken to ensure that peak absorption were devoid of contaminants with absorption overlapping detection wavelength [75].

In our knowledge, the only quantitative approach for HPLC-MS analysis of MAAs was published by Whitehead and Hedges [148]. The authors used a LC/MS comprising a Shimadzu HPLC with a dual wavelength detector interfaced to a Micromass Quattro II (QHQ) mass spectrometer (Micromass Manchester, UK) system that was calibrated with seven pure MAA standards. Their results showed that each MAAs exhibited a different sensitivity to ionization. Shinorine and porphyra-334, two higher polarity MAAs, were the least sensitive compounds while the less polar, palythine and palythene were most easy ionized. Their results also showed that quantitative mass spectral analysis of MAAs standards compared to a L-tyramine hydrochloride (THC) internal standard have a precision of better than 3% and a detection limit of ∼2.0 pg. Detection of the MAAs by extracting the [M + H]^+^ ion from the total ion current scans (TIC) was estimated ∼100 times more sensitive than by UV absorption. However, this sensitivity could likely be improved by a factor of 10–100 if selective ion monitoring (SIM) were used rather than a full ion scan [148]. In this method, LC separation of MAAs was based on the low resolution reverse phase HPLC approach outlined by Dunlap *et al.* [61] and in consequence they can not distinguish geometric MAA isomers such as palythene/usujirene and *E/Z*-palythenic acid. A major problem is that crude methanolic extracts without any sample clean-up were directly injected to the HPLC. It is widely accepted that in both ESI and APCI, the ionization rate of the analytes depends strongly on the physicochemical environment in the ion source. ESI is reported to be more susceptible to subtle changes in the characteristics of the LC-effluent than APCI. However, in both methods, samples with a complex matrix like biological extracts can cause MS signal suppressions or enhancements, which are termed “matrix effects” [157]. It is widely believed that these effects are due to ionization competition between different species eluting from the HPLC column. Matrix effects are generally not reproducible, or repeatable between various samples or even between different injections of the same sample and, thus, can severely compromise quantitative analysis [156]. Matrix effects can be minimized by improving the sample preparation to achieve as clean as possible extracts, by optimizing the chromatographic procedure to separate the analytes from the matrix effects, by changing the ionization conditions, or by a combination of the above. However, the most efficient way to circumvent matrix effects is the use of stable-isotope labeled internal standard [158]. Both the signal intensities of the analyte and its stable isotope labeled analogue were influenced by any matrix effects in the same way, leaving the ratio between them constant and therefore a reliable quantification can be achieved despite any matrix effects.

### 2.4. Occurence of MAAs in Marine Organisms

The mycosporine-like amino acids (MAAs) have been found in cyanobacteria [20,56,74,159], red algae [74,91,160], phytoplankton [10,111,112], lichens [74], gorgonians [161], corals and their associated biota [61,142,147,162–164], as well as in many other marine organisms such as other cnidarians [85–87,104,165], sponges [101,146], brine shrimp [166], sea urchins [24,167], starfish [146], holothurids [168], clams [169], ascidians [170,171] and fish [141,172,173]. Recently a database on distribution of mycosporine and mycosporine-like amino acids in marine and freshwater organisms has been developed [70].

Although these data are very valuable, it is necessary to keep in mind that in most cases, mycosporine-like amino acids have been identified by co-chromatography with sub-standards and/or by comparison with published UV spectral data and HPLC retention times. Therefore, such characterization of these compounds should be treated with caution [11,17,99]. For instance, mycosporine-glycine-valine has been partially characterized (see Table 1) from the Antarctic pteropod *Limacina helicine* [74] and their extracts used in many cases as sub-standards. However, posterior LC-MS/MS analysis of this extracts and of other Antarctic pteropods, revealed the presence of palythenic acid in place of mycosporine-glycine-valine [99]. The same controversial results could be observed in the MAAs composition reported for the Antarctic krill *Euphausia superba* [146,174]. In addition, many MAAs still have to be fully characterized [34,75,85,111–114]. A continuous survey of organisms and methodological advances [75,78,79,148] may reveal new MAAs and hence, the diversity and distribution of these compounds may be greater than is presently recognized.

### 2.5. MAAs in Producing Organisms

#### 2.5.1. Biosynthesis of Primary MAAs

**Figure 6.**
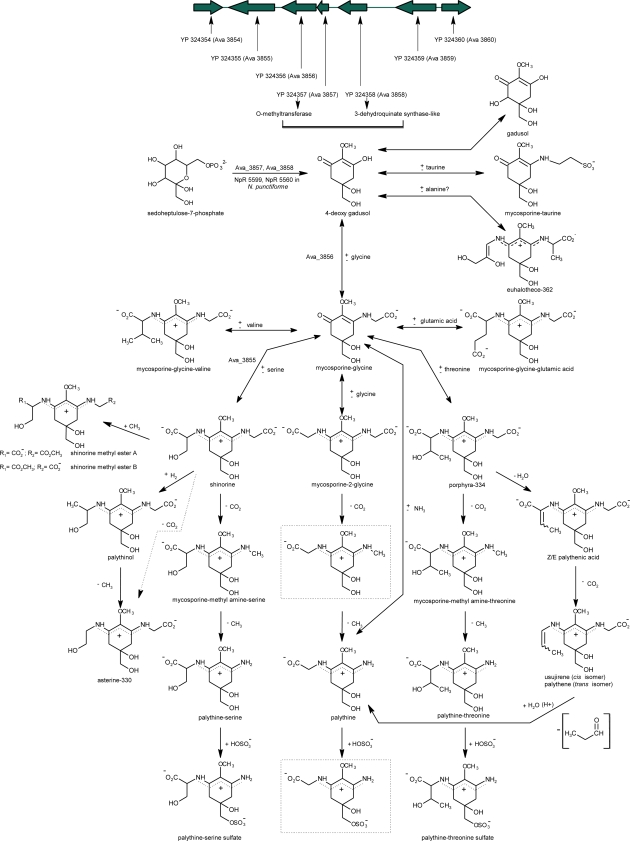
Structural relationships between the different mycosporine-like amino acids, their feasible biochemical conversions (modified from Carreto *et al.* [75]) and the proposed role of the genes. Ava_3858, Ava_3857, Ava_3856 and Ava_3855 isolated from *A. variabilis* PCC 7937 (ATCC 29413). (Adapted from Singh *et al.* [73] and Balskus and Walsh [102]).

According to Favre-Bonvin *et al.* [82], the synthesis of mycosporines in the deuteromycete *Trichothecium roseum* would have its origin in the route of shikimate. These authors showed that the precursor of the six-carbon ring common to all mycosporines in fungi was 3-dehydroquinate (3-DHQ). The synthesis of fungal mycosporines would presumably proceed from 3-DHQ through gadusol or deoxygadusol, possibly being these last two compounds the precursors of mycosporine-glycine. The blockage of the MAAs synthesis in the coral *Stylopora pistillata* produced by the addition of a shikimate route inhibitor (glyphosate = *N*-phosphonomethyl-glycine) provided the first direct evidence of the MAAs synthesis starting from this route in marine organisms [19,147]. More recently, Portwich and Garcia-Pichel [175] have suggested that the central ring of the MAAs present in the cyanobacterium *Chlorogloeopsis* sp. strain 6912 has its origin in the shikimate pathway, suggesting that the biosynthesis of the MAAs would be very similar or possibly identical in eukaryotic and prokaryotic organisms. However, the recent results of Balskus and Walsh [102] contradict the longstanding assumption that MAA biosynthesis involved a Shikimate pathway intermediate. These authors failed to observe any 6-deoxygadusol production when the putative substrate 3-dehydroquinate was incubated with the dehydroquinate synthase (DHQS) and O-methyltransferase (O-MT) homologues (NpR 5600 and NpR 5599 respectively) from *Nostoc punctiforme* (ATCC 29133) and the typical cofactors S-adenosylmethionine (SAM), nicotinamide adenine dinucliotide (NAD^+^) and Co^2+^. In addition, treatment of the intermediate of the pentose phosphate pathway: sedoheptulose 7-phosphate (SH7-P) with NpR 5600 and NpR 5599 in the presence of SAM, NAD^+^ and Co^2+^, produce a single product, 6-deoxygadusol. In their experiments, using radiolabeled amino acids, Portwich and Garcia-Pichel [175] detected for the first time the specific incorporation of ^14^C-glycine and ^14^C-serine into the corresponding side chains of mycosporine-glycine and shinorine, demonstrating that these free amino acids are their direct precursors and that mycosporine-glycine is the direct metabolic precursor of the MAA bisubstituted shinorine. Similar results were observed recently in the cyanobacterium *Anabaena doliolum*. In this cyanobacterium, mycosporine-glycine acts as precursor for the biosynthesis, induced by UVB radiation, of shinorine and porphyra-334 [73,176]. MAA biosynthesis in this organism is dependent on photosynthesis only for the carbon source, since the inhibitory effect of DCMU on MAA biosynthesis [10] was overcome by externally added fructose [176]. These and other evidences [73,75,109,163,177] support the idea that the mycosporine-glycine condensation with an amino acid would be a common reaction in the generation of amino acid bi-substituted MAAs, since most of them contain a glycine residue. The results of Balskus and Walsh [102], confirms that the expression of the gene Ava_3856 from *Anabaena variabilis* can convert 6-deoxygadusol and glycine into mycosporine-glycine in the presence of Adenosine Triphosphate (ATP) and Mg^2+^ cofactors. These authors also confirmed the biosynthetic role of a nonribosomal peptide synthetase (NRPS)-like enzime Ava_3855, in the ATP-dependent conversion of mycosporine-glycine and serine to shinorine (Figure 6). In contrast, nothing is known about the chemically plausible condensation of amino acids with mycosporine-taurine, the other known oxo-carbonyl MAAs.

#### 2.5.2. Biosynthesis Regulation

Carreto *et al.* [65,178] were the first to show an increase in PAR irradiance resulted in increased synthesis of MAAs and that UVA (315–400 nm) and blue light stimulated the synthesis of MAAs in free-living dinoflagellates. After this, similar results were found for several species of dinoflagellates [23,110,177,179,180]. However, UVB wavelengths (280–315 nm) were more effective to induce MAAs accumulation in the dinoflagellate *G. dorsum* [178]. In Antarctic diatoms, UVA and blue light were effective to induce MAAs synthesis [181–183], while UVB + UVA were more effective for prymnesiophytes [181,182]. In cyanobacteria, MAAs can be induced by PAR, UVA and UVB radiation [184]. However, UVB has the most pronounced effect in comparison with the other wavelength ranges [184,185]. Corals needed a combination of UVB and UVA in addition to PAR to stimulate the synthesis [147,186]. Hoyer *et al.* [187] stressed the fact that no consistent induction pattern could be found for red macroalgae from Antarctica. They observed three induction patterns among the 8 out of 18 species that showed an induction of MAAs: (1) responding to the full radiation spectrum; (2) responding to PAR + UVA, with no effect of additional UVB; and (3) similar to (2) except that additional UVB caused a decrease in MAAs. Hence it may be premature to extend results of the previous studies to whole group patterns. Physiological acclimation and adaptation may play a greater role than previously suspected [110,183,188,189].

The wavelength dependence for MAA synthesis indicates that specific photoreceptors are present [179,190–192]. However, these have not yet been identified. Kräbs *et al.* [192] determined the action spectrum for the synthesis of MAAs in the marine red macroalgae *Chondrus crispus*. They suggested an unidentified UVA-type photoreceptor with a major absorption peak at 340 nm, a second maximum at 320 nm and a smaller third peak at 400 nm. Other suggestions for UVB photoreceptors include a reduced pterin in cyanobacteria based on action spectra for MAA induction peaking at 310 nm [193]. Shick [163] discussed that there may be no need for a specific UVB photoreceptor, if the primary response to UVB is via ROS (reactive oxygen species) and the induction of MAAs responds to the increases in ROS. Once this level is reached, ROS would accumulate and they would secondarily control the synthesis of MAAs, as suggested by Shick [163]. Other stress factors than PAR or UV radiation have been found to stimulate the synthesis of MAAs.

Osmotic stress, alone or in combination with UVB radiation, can induce the synthesis of MAAs in some cyanobacteria [184,194]. However, as has been previously discussed [183,189] UVB radiation and other stress factors can modulate the accumulation process of MAAs by modifying the cellular balance between rates of net synthesis, excretion [195] and cellular division. For instance, during the initial days of acclimation to full solar radiation, the cellular division of the diatom *Thalashiossira* sp. [183] and the dinoflagellate *Alexandrium catenella* [189] were strongly inhibited by UVR. As the synthesis of MAAs was not affected by UVR, the cell exposed to full solar radiation accumulated more MAAs that in the PAR treatment. This probably means that cells have to invest a significant amount of energy in the synthesis of photoprotective compounds and repair mechanisms at the expense of the build-up of carbon skeletons. As can be expected, at higher UVB exposure (BED_DNA_ 305_nm_ = 2.78 kJ·m^−2^·d^−1^), cell division and MAAs production in *Alexandrium tamarense* were totally blocked [189]. In a recent study on growth and photoprotection in two strains of *A. tamarense* [110], the highest cell concentrations of MAAs were associated with the strain that showed greatest signs of stress, and this response was clearly associated with the UVB dose. Moreover, highly UVB-stressed cells favored the accumulation of secondary MAAs such as shinorine methyl ester and palythine, while less-stressed cells showed more primary MAAs (notably more of the antioxidant mycosporine-glycine) in relative proportion, and maintained higher constitutive levels of those after the recovery period [110].

There are pronounced effects of nitrogen supply on the synthesis of MAAs. In the red macroalga *Porphyra columbina*, addition of ammonium to the medium combined with different irradiation treatments led to increased MAA concentrations based on dry weight [50]. In the cyanobacterium *A. variabilis* PCC 7937, MAA synthesis was favored by ammonium in a concentration-dependent manner, without or in combination with UV stresses [184]. On the contrary, nitrogen limitation significantly decreased the synthesis of MAAs in the dinoflagellates *Akashiwo sanguinea* and *Gymnodinium cf. instriatum* [196]. The MAAs composition is also highly influenced by nitrogen status. In the dinoflagellates *A. sanguinea* [196], *A. tamarense* and *Karenia brevis* the N-starved cells have much higher percentage of mycosporine-glycine [197]. This MAA is the least N-rich among all MAAs found in these dinoflagellates, and offers protection in the most harmful part of the spectrum [196]. MAAs and toxins are secondary metabolites [198] with no essential functional role, thus under N-limitation, energy and intra-cellular pools of nitrogen would mainly be allocated to the maintenance of basic and essential cellular functions, and the activation of energetically costly and nitrogen demanding metabolic pathways, such as MAAs synthesis, would not be favored. In toxic *Alexandrium* strains, depletion of nitrogen also causes PSP toxin content to decrease [199,200] in parallel with other metabolic changes that occur during N-stress, whereas under P-limited conditions and growth at suboptimal temperatures, toxin content increases dramatically [198]. In our knowledge, there is not available information on MAAs synthesis and accumulation under P-limited conditions and growth at suboptimal temperatures. Recently, Singh *et al.* [201] showed for the first time that synthesis and bioconversion of a primary mycosporine-like amino acid (MAA) into a secondary MAA is regulated by sulfur deficiency in the cyanobacterium *A. variabilis* PCC 7937.

Independently of the induction mechanisms, response kinetics seem to vary substantially among organisms, with a rapid response time for some, within the first few hours of exposure to higher irradiances in dinoflagellates [10,13,109,110,177,180] and a much slower response for others; several days to weeks in cyanobacteria [20,184], corals [163] and macroalgae [202].

#### 2.5.3. MAAs Distribution

Details of the pathway and the enzymes involved in the biosynthesis and transformations of different MAAs in cyanobacteria, marine algae and phototrophic symbiotic organisms are still unknown and remain a topic of discussion. Nevertheless, it has been assumed that the high diversity of MAAs present in marine organism is principally derived from the synthesis and transformation of mycosporine-glycine, mycosporine-2-glycine, shinorine and porphyra-334 [10,109,147,163,176,179,184,201,203,204] (Figure 6). This indicates the evolutionary significance of these compounds during the course of evolution by conserving them in a great diversity of marine organisms, in contrast to scytonemin which is only limited to cyanobacteria [20]. Notably, derivatives from the disubstituted amino acids, mycosporine-glycine-valine and mycosporine-glycine-glutamic acid were not reported.

##### 2.5.3.1. Cyanobacteria

There are reports that some *Anabaena* spp. only synthesize the MAA shinorine [20,43,70] while other *Anabaena* spp. isolated from the same habitat lack the ability to synthesize this and other MAAs [73,129,205]. Recently four cyanobacteria, e.g., *A. variabilis* PCC 7937, *Anabaena* sp. PCC 7120, *Synechocystis* sp. PCC 6803 and *Synechococcus* sp. PCC 6301, were tested for their ability to synthesize MAAs, and genomic and phylogenetic analysis was conducted to identify the possible set of genes that might be involved in the biosynthesis of these compounds. Out of the four investigated species, only *A. variabilis* PCC 7937 was able to synthesize MAA [73,176]. However, whereas these authors were not able to detect any trace of MAAs from *Synechocystis* sp. PCC 6803, Zhang *et al.* [34] have reported the presence of some unusual and new MAAs from this cyanobacterium (mycosporine-taurine, dehydroxylusujirene), different from the normally reported MAAs (asterina-330, euhalothece-362, mycosporine-glycine, palythene, palythinol, porphyra-334 and shinorine) in cyanobacteria [20,70]. However, as has been previously noted by Singh *et al.*, [73] the identity of these compounds is questionable. Moreover, genomic analysis of four fully sequenced cyanobacteria identified a combination of genes that might be involved in MAA biosynthesis: YP_324358 (predicted DHQ synthase) and YP_324357 (O-methyltransferase), which were present only in *A. variabilis* PCC 7937 and were missing in other studied cyanobacteria, including *Synechocystis* sp. PCC 6803 [73]. On the other hand, phylogenetic analysis revealed that these two genes are transferred from a cyanobacteria donor to dinoflagellates and finally to metazoan [72,73] by lateral gene transfer. Singh *et al.*, [73] also suggest that the predicted protein structure for YP_324358 is different from the chemically characterized DHQ synthase of *Aspergillus nidulans* [206], contrary to the YP_324879, which was predicted to be similar to the DHQ synthase of *A. nidulans*. Based on these results Singh *et al.*, [73] proposed that the YP_324358 and YP_324357 gene products are involved in the biosynthesis of the common core (deoxygadusol) of all MAAs (Figure 6). Recently, Balskus and Walsh [102] reported the identification of a MAA biosynthetic gene cluster in *A. variabilis* ATCC 29413 and the discovery of analogous pathways in other sequenced organisms.

The MAA signature for most cyanobacteria able to synthesize these compounds consists of shinorine, porhyra-334 and in some cases mycosporine-glycine [20,70,73,77,176]. For example, among *Anabaena* spp., the MAA profile of a rice-field cyanobacterium, *A. doliolum*, is unique as it reveals the biosynthesis of three MAAs, mycosporine-glycine, porphyra-334 and shinorine [73,176]. Three species of *Nodularia* (*N. baltica*, *N. harveyana* and *N. spumigera*)—a filamentous and heterocystous cyanobacterium from the Baltic Sea—synthesized shinorine and porphyra-334 [207,208]. The photo-oxygenic prokaryotic *Prochloron* sp., a symbiotic cyanobacterium exclusively found in association with tropical colonial ascidians, also has been reported to contain high amounts of MAAs. Isolated *Prochloron* cells from *Lissoclinum patella* contained shinorine, which was also dominant in the host tunic together with minor amounts of mycosporine-glycine and palythine [170]. The same species was reported to contain shinorine and mycosporine-glycine [27] or only mycosporine-glycine [209]. However, some cyanobacteria groups contain multiple MAAs, but with very few exceptions their structures have not been elucidated. Among all the *Microcoleus* (Oscillatoriales) strain studied by Karsten and García-Pichel [210] four different MAAs were detected, one of them shinorine and three unidentified compounds showed absorption maxima at 332, 344 and 346 nm. Recently, mycosporine-alanine, mycosporine-glutaminol, mycosporine-glutaminol-glucoside, previously described in terrestrial fungi, were found in terrestrial cyanobacteria (*Oscillatoriales*) [76,78] In addition, mycosporine-2-glycine and a rare novel MAAs containing the amino acid alanine (euhalothece-362) was described in the unicellular cyanobacterium *Euhalothece* sp., inhabiting in a hypersaline saltern pond [77,100]. The MAAs such as asterina-330, shinorine, palythinol and mycosporine–glycine, have been reported to play a photoprotective role in epilithic cyanobacteria from a freshwater lake [211]. The freshwater bloom forming cyanobacteria *Microcystis aeruginosa* also contain high concentrations of MAAs including shinorine and several unknown MAAs [114]. The diazotrophic ocean bloom forming cyanobacteria *Trichodesmium* spp. were also reported to contain high amounts of MAAs. Among those MAAs identified, asterina-330 and shinorine were the most abundant. Also found were mycosporine-glycine, porphyra-334 and palythene. However, two of the most abundant compounds were unidentified [113]. There is however a dearth of studies of MAAs in oligotrophic oceans where prokaryotes are known to dominate [212]. Samples collected at the surface of Brazil Current where *Synechococcus* spp. and *Prochloroccocus* spp were the major components of the phytoplankton assemblage, showed the higher MAAs/chlorophyll *a* (chl *a*) [213] of the studied area [214] and a complex MAAs composition that includes shinorine, palythine, porphyra-334, mycosporine-glycine, palythenic acid and shinorine-methyl ester [75].

Details of the pathway and the enzymes involved in the bio-transformations of primary MAAs in cyanobacteria remain to be elucidated. However, recently bioconversion of a primary MAAs into a secondary MAA was found to be regulated by sulfur deficiency in the cyanobacterium *A. variabilis* PCC 7937 [176,201]. This cyanobacterium synthesizes the primary MAA shinorine under normal conditions, however under sulfur deficiency, a secondary MAA palythine-serine appears. Addition of methionine to sulfur-deficient cultures resulted in the disappearance of palythine-serine, suggesting the role of primary MAA under sulfur deficiency [201]. This is the first time that palythine-serine was found to be synthesized by cyanobacteria which had so far been reported only in corals [7,75,79,84,142,147,164]. Addition of methionine also affected the steady state between mycosporine-glycine biosynthesis and its conversion into shinorine, consequently, resulting in the appearance of mycosporine-glycine [201]. Results also support that palythine-serine is synthesized from shinorine after decarboxylation and demethylation of the glycine subunit as proposed earlier [75,79,215]. Based on these results, Singh *et al.* [201] suggests that glycine decarboxylase is the enzyme that possibly catalyzes the decarboxylation and demethylation of the glycine unit of shinorine and subsequently transfers the methyl group to tetrahydrofolate that ultimately participates in the regeneration of methionine under sulfur deficiency (Figure 6).

##### 2.5.3.2. Microalgae

Marine algae lineages appear to have arisen through a secondary endosymbiosis between a heterotrophic flagellate that engulfed a single-celled red or green algae, which itself traces back to a primary endosymbiotic event in which a heterotrophic protist engulfed a cyanobacterium [216]. For instance, in the dinoflagellates *Heterocapsa triqueta* and *Oxyrrhis marina*, a dehydroquinate synthase (DHQS) similar toYP_324358 of *A. variabilis* PCC 7937 [201] has been reported to be present in the chloroplast and to be fused to O-methyltransferase [217]. However, in *Karlodinium micrum* both DHQS and O-methyltransferase are not fused proteins but the O-methyltransferase is present downstream of the DHQS (dehydroquinate synthase) in the same reading frame. In the dinoflagellates *H. triquetra*, *O. marina* and *K. micrum* both genes have been reported to be transferred from cyanobacteria via a prokaryote-to-eukaryote lateral/horizontal gene transfer event during evolution [201,217]. Therefore, genes derived from both photosynthetic and heterotrophic lineages may account for differences in the biosynthesis, accumulation and conversions of MAAs among marine algae.

**Figure 7.**
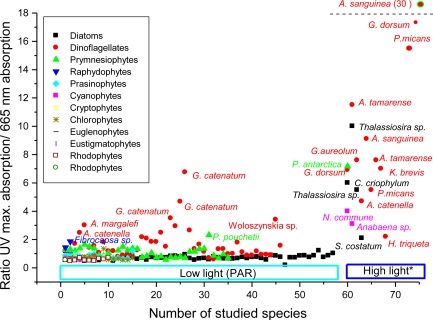
Relative abundance of MAAs respect to chlorophyll *a*, estimated by the absorbance ratio UV λ_max_/λ = 665 nm measured on methanolic extracts from several species of marine phytoplankton growing under low light PAR, and under high light * (PAR, or PAR complemented with UVA or UVA + UVB). Data were compiled from [10,13,23,26,65,66,111,112,149,174,177,183,196,197].

The most comprehensive study (152 species) on the abundance of total UV-absorbing compounds in microalgae showed that high levels of these compounds were found in some dinoflagellates, cryptophytes, prymnesiophytes and raphidophytes, with highest levels in surface bloom forming dinoflagellates (Figure 7).

Lower levels were reported in diatoms, chlorophytes, euglenophytes, eustigmatophytes, rhodophytes, some dinoflagellates and some prymnesiophytes [112]. Lellewing and Airs [111] arrived to a similar conclusion in their study on the composition and abundance of MAAs in a diverse range of microalgal cultures (33 species across 13 classes). However, in both studies cultures were grown under low irradiance fluorescent “daylight”, without supplementary UV. Therefore, light composition may account for differences observed in the biosynthesis and accumulation of MAAs among the studied microalgae (Figure 7).

###### 2.5.3.2.1. Dinoflagellates

The earliest studies noting high UV absorbance in marine phytoplankton were developed in dinoflagellates [63,64]. However, the chemical nature of these UV-absorbing substances in dinoflagellates (MAAs) was not known until the paper of Carreto *et al.* [10]. From there on, many studies provide evidence that the commonly occurring symbiotic dinoflagellate *Symbiodinium*, can synthesize MAAs [14,163,164,218–221]. However, all species of symbiotic dinoflagellates do not synthesize MAAs. For example, MAAs were neither detected in *Symbiodinium californium* in culture medium, nor in the algae freshly isolated from *Anthopleura elegantissima* [14,218]. Recently, Banaszack *et al.* [164] showed that regardless of the clade identity, all natural *Symbiodinium* spp. in cnidarian host from the Mexican Caribbean contain MAAs, in contrast to the pattern that has been found in cultures of *Symbiodinium*, where clade A symbiont produced MAAs whereas clade B, C, D, and E symbionts did not [221]. Under natural conditions, between one and five MAAs were identified in the symbiont fractions, mycosporine-glycine, shinorine, porphyra-334, mycosporine-2-glycine and palythine [163,164,221]. As has been demonstrated for cyanobacteria [184], there is an order of appearance of MAAs in *Symbiodinium*: mycosporine-glycine followed by shinorine then porphyra-334 and finally palythine [164].

Some photosynthetic free-living species of dinoflagellates such as *Prorocentrum minimun* [43] and *Woloszynskia* sp. [112] also contain a few MAAs whereas in *Amphidinium carterae* only mycosporine-glycine was found [182]. Contrarily and consistent with previous findings [10] highest levels and diversity of MAAs are among surface bloom forming dinoflagellates [10,11,13,14,23,26,43,75,109–112,115,116,148,153,177,180,195–197,220]. Red tide dinoflagellates such as *A. sanguinea* (=*Gymnodinium sanguineum*) contain several primary MAAs (mycosporine-glycine, shinorine, porphyra-334) as well as palythine and palythene [23,196] and *Alexandrium* species also contain asterina-330, palythinol, palythenic acid, usujirene, palythene, shinorine methyl ester and other unnamed MAAs [10,11,75,109–111,115,116,148]. Interestingly, the red tide dinoflagellate *P. minimum* showed the net dominance of shinorine methyl ester together with less amounts of shinorine, palythine, mycosporine-glycine, palythenic acid, palythene, usujirene and two unidentified MAAs [153] also observed in *Alexandrium* spp. [11]. On the other hand, *K. brevis* has a simple MAAs profile mainly composed of mycosporine-glycine with some shinorine, porphyra-334 and a small amount of palythinol [197]. Recently, a total of seven MAAs were identified in two strain of *A. tamarense* and *H. triqueta*: mycosporine-glycine, shinorine, porphyra-334, palythenic acid, palythine, usujirene, palythene and a major unknown MAAs that correspond most likely to shinorine methyl ester [110]. According to the authors, other unknown MAAs included the putative mycosporine-taurine [110]. A similar unknown compound with a λ_max_ at 310 nm was also found in high concentrations in the dinoflagellate *Scrippsiella trochoidea* [111]. They also observed two novel MAA-like compounds with distinct and previously unreported absorption maxima in the dinoflagellates *Gymnodinium galatheanum*, *Gymnodinium veneficum* (λ_max_ = 342 nm) and *Prorocentrum micans* (λ_max_ = 352 nm). It is interesting that unknown MAA compounds with similar absorption maxima (at 346 and 342 nm) have been described before in several species of the cyanobacterial genus *Microcoleus* (Oscillatoriales) [210]. Compounds with λ_max_ > 360 nm have been also observed in the dinoflagellate *Gymnodinium catenatum* [75,112] and in the Euglenophyte *Euglena gracilis*, in the raphidophyte *Heterosigma akashiwo* and in the rhodophyte *Porphyridium purpureum* [111]. Further detailed chemical structural analysis using mass spectrometry and nuclear magnetic resonance is required to identify these compounds.

Although a high diversity of MAAs was detected in *A. tamarense* [11,110,197], differences in MAAs content and composition between strains can sometimes be as important as differences between species or algal groups [11,110,153,197]. Large intrastrain differences have also been reported for toxin (saxitoxin congeners) composition of *A. tamarense* isolates [199,222,223]. Moreover, the toxin concentration and toxin composition of natural populations of *A. tamarense* were more homogeneous and very different to those of nutrient-replete cultured strains [199]. These results indicate that there is a clear need for further investigation on how culture conditions vary the metabolism of these secondary metabolites [199] in response to cultivation, or how environmental conditions select the more adapted strains. Recently, Moustafa *et al.* [224] provided evidence that the assembly of STX genes in the cyanobacteria *Anabaena circinalis* involved multiple horizontal gene transfers (HGT) events from different sources followed presumably by coordination of the expression of foreign and native genes in the common ancestor of STX + cyanobacteria. Therefore, although it is unknown if dinoflagellates gain the machinery via a single or multiple HGT events from MAAs + cyanobacteria, it is clear that actually, the interpretation of MAAs contents variability between cultured strains is considerably difficult. Another possible explanation for the high diversity of MAAs observed in bloom-forming dinoflagellates is the role than other organisms—most notably bacteria—can play in MAA synthesis. It is feasible that endosymbiotic bacteria present in some of these organisms [225] are capable of the *de novo* synthesis of MAAs [226] or more probably, they may be responsible for some interconversions among primary and secondary MAAs [11]. For instance, the variable kinetics of increase among these MAAs in *Alexandrium*, where the MAAs complement may change on the order of hours, indicate rapid inter-conversions among different MAAs [10,11,100,177,197] (Figure 6). Carreto *et al.* [10] suggested that porphyra-334 could be the precursor of palythenic acid and that this compound could get transformed to usujirene and palythene. Taira *et al.* [177] observed a daily variation in cellular content and in the relative composition of MAAs during the cell cycle of the dinoflagellate *Scrippsiella sweeneyae*. The highest relative abundance in mycosporine-glycine at the beginning of the light period, and the successive increase of shinorine and phorphyra-334 within the first 6 h of light exposure, support that mycosporine-glycine is the direct metabolic precursor of primary MAAs [19,109,175,227]. On the other hand, the decrease in the relative abundance of shinorine and porphyra-334 during the dark period with the concomitant increase in the relative abundance of palythine -and in minor proportion that of palythene- indicate that inter-conversions between shinorine and palythine might occur within a few hours [177]. More recently, Callone *et al.* [109] observed a two-stage process for the induction of MAAs in *A. tamarense*. These authors showed an increase in primary MAAs, mycosporine-glycine and notably porphyra-334, within the first 2 h of exposure to higher PAR irradiances, after which they decreased and were replaced by increasing levels of *Z*-palythenic acid, usujirene, palythene and shinorine methyl ester and minor amounts of other unusual complex MAAs named as M-320, M-335/360 [11]. Even if there is not direct evidence provided from the utilization of labeled compounds, it is probable that usujirene and palythene in *Alexandrium* may be formed via dehydration of porphyra-334 (*Z*-palythenic acid) followed by decarboxylation (usujirene) and isomerization of the formed usujirene [10,11,75,109,123]. On the other hand, in *A. tamarense* shinorine appears to be the precursor of shinorine methyl ester and M-320 [109]. The chemical structure of shinorine methyl ester was not completely elucidated, but the proposed transformations are in accordance with the chemical structures of shinorine (Figure 6). The monomethyl ester of shinorine appeared to be synthesized from shinorine by preferential esterification of one of the two carboxyl function of shinorine [228], while M-320 would result from the condensation of shinorine with mycosporine glycine [11,109]. Heterotrophic dinoflagellates are believed to be unable to synthesize MAAs. However, Llewellyn and Airs [111] found that the heterotrophic dinoflagellate *O. marina* contain MAAs at similar concentrations as in other dinoflagellates and exceeding those of the diatom cultures analyzed. This suggests that the MAAs in *O. marina* did not originate from the diatom food source and were possibly synthesized. Notably, *O. marina* contains genes encoding shikimic acid pathway enzymes (HQ-Synthase and *O*-methyltransferase) obtained by horizontal gene transfer from a cyanobacterial source [73,217].

###### 2.5.3.2.2. Prymnesiophytes

MAAs were found in abundance in several prymnesiophyte species and in particular in species of *Phaeocystis* from Antarctica. *Phaeocystis pouchetti* from Antarctica as being inherently high in UV absorbing compounds and increases its concentration in response to exposure to PAR [229,230] and PAR + UV-radiation [174,229]. Concentration of MAAs in *P. pouchetti* appeared to be dependent on strain, stage in life cycle and the presence of bacteria [229]. Individual MAAs, including the tentatively identified mycosporine-glycine-valine (probably palythenic acid), has been found to be present in *P. pouchetti* accompanied by the more prevalent shinorine and mycosporine-glycine [174,230]. Other species of prymnesiophytes including the near ubiquitous species *Emiliania huxleyi* have been shown to contain a variety of MAAs although identification of specific compounds is often tenuous [182]. In the bloom-forming coccolithophorid *E. huxleyi* only shinorine was present in quantifiable amounts, whereas palythine and porphyra-334 were present at trace level [75]. The inability of *E. huxleyi* to accumulate MAAs could possibly be related to this species’ poor tolerance of ultraviolet radiation [231]. Of the three species of prymnesiophytes examined by Llewellyn and Airs [111], *Isochrysis galbana* and *E. huxleyi* contained MAAs at low concentrations and *Phaeocystis globosa* contained no detectable MAAs.

###### 2.5.3.2.3. Raphidophytes

Similar to dinoflagellates and prymnesiophytes, high levels of MAAs were found in surface bloom forming raphidophytes. The rhaphidophyte species *Heterosigma carterae* and *Fibrocapsa* sp. have high levels of MAAs with both containing the more unusual secondary MAA asterina-330 [111,112]. As in the case of dinoflagellates, differences between strains can sometimes be as important as differences between species or algal groups. For instance, an Australian strain of the raphidophyte *Chattonella marina* produced five times more MAAs (mycosporine-glycine; mycosporine glycine-valine and shinorine) and grew 66% faster than a Japanese strain of the same species under inhibiting UVB radiation [232]. Increased MAA production under high irradiance was also observed in other Australian strains of *Chattonella*, but not noted in other Japanese strains suggesting ecophenotypic adaptation due to differing environmental conditions [232].

###### 2.5.3.2.4. Diatoms

Constitutive levels of MAAs are generally lower in cultured diatoms compared to dinoflagellates [111,112], although this may differ for natural populations exposed to high radiation levels. The synthesis of MAAs was not part of the UVB response in several studied diatoms [110,115,181], in contrast to some other studies [182,188], notably with Antarctic diatoms [74,149,183]. Nine bacillariophyte species within one study were all reported to contain predominantly the primary MAAs porphyra-334 (75–95%) with lesser amounts of shinorine (5–20%) and mycosporine-glycine additionally present in one species, *Porosira pseudodenticulata* [181]. Six different species of *Thallasiosira*, primarily from Antarctica have been investigated for MAA contents and were found to be either absent in MAAs or to contain shinorine and porphyra-334 [74,115,149,183] with only mycosporine-glycine identified in another study [182]. Although the MAA signature for most diatoms consists on primary MAAs (porhyra-334, shinorine and mycosporine-2 glycine) the presence of the secondary MAAs palythine and palythene has been reported in *Corethron criophilum* [149]. In addition, Carreto *et al.* [75] based on co-elution with mycosporine-taurine from *A. elegantissima* and with published UV spectral data and HPLC retention times, have reported the presence of the unusual mycosporine-taurine in *Pseudo-nitzschia multiseries*. This is the first time that mycosporine-taurine was found to be synthesized by a diatom which has been so far reported only in sea anemones [85–87] but recently also tentatively identified in dinoflagellates [110]. A recent study of diatom frustules-bound organic matter in opal-rich Southern Ocean plankton and sediments revealed the presence of several bound or entrapped MAAs in the frustules of marine diatoms. Palythine, porhyra-334 and shinorine were the most abundant MAAs detected in fluorhydric acid digests of plankton and sediment. Traces of asterina-330, palythinol and palythenic acid was also detected [120]. The authors hypothesize that the mineral matrix could stabilize these compounds and suggest the possibility that they could be used as indicators of past solar irradiance.

###### 2.5.3.2.5. Other Algae Groups

Species analyzed within the chlorophytes, eustigmatophytes, prasinophytes and rhodophytes lacked commonly occurring MAAs although several species contained minor amounts of unknown UV absorbing compounds [111,112,182,233]. Xiong *et al.* [233] were the first to detect UV-absorbing compounds as well as their induction and accumulation after UV exposure in a sample of six coccoid green algae from freshwater phytoplankton, but the exact nature of these UV-absorbing compounds has not been determined in their study. Only of few marine green microalgae produce MAAs. *Dunaliella tertiolecta* (Chlorophyceaea) and *Pyraminonas parkerae* (Prasinophyceae) have been reported to contain the tentatively identified MAAs mycosporine-glycine [182]. However, Sonntag *et al.* [234] were able to confirm the synthesis of several MAAs (mycosporine-glycine, shinorine, porphyra-334 and palythine) by symbiotic *Chlorella* isolated in culture from *Chlorella*-bearing ciliates, thus providing initial evidence for their symbiotic origin. Recently MAAs designated as 322 nm-MAA and 324nm-MAA were detected within a distinct clade of the Trebouxiophycea of the Chlorophyta [235]. These compounds were also detected in aeroterrestrial film algae isolated from building facades. Their function as sunscreen was well supported [236].

###### 2.5.3.2.6. MAAs in Natural Phytoplankton Populations

The observation of a peak in the UV range (near 330 nm) in *in vivo* absorption spectra for near surface waters dates back to the early 1980s [58,59]. At about the same time, Carreto *et al.* [65] and Vernet *et al.* [66] observed that red-tide populations of dinoflagellates (*Gonyaulax excavata = A. tamarense*, *P. micans*, and *Gonyaulax polyedra* = *Lingulodinium polyedra*) produce MAAs in abundant quantity.

In Antarctica, high *in vivo* absorption at wavelengths indicative of MAAs, was characteristic of assemblages dominated by prymnesiophytes [237] and by the chain-forming diatom *Thalassiosira gravida* [238]. The presence of MAAs in these organisms was clearly related to the UV peak observed in *in vivo* spectra, which reflects the combined influence of the various MAAs present. This UV peak was often several times higher than the red absorption peak of the chlorophyll *a*. Moisan and Mitchell [230] and Frame [197] reported values of chlorophyll-specific phytoplankton absorption at 330 nm 4 to 13 times greater than the absorption at 676 nm. Assemblages dominated by *Phaeocystis antarctica* also had very different, more complex, MAAs profile (mycosporine-glycine, shinorine, porphyra-334, palythine, palythinol and palythenic acid) from those dominated by diatoms [197]. In diatom-dominated assemblages, mycosporine-glycine was always abundant and occasionally porphyra-334 and shinorine were present.

Whitehead and Vernet [239] followed extracted MAAs as well as UV absorption in particulate and dissolved material between March 1995 and April 1996 in La Jolla Bay, California. Their study was the first to actually extract and determine the concentration of MAAs from the dissolved pool in seawater. Maximum particulate UV absorption was observed in the spring, following a large red tide of *L. polyedra*, while maximum dissolved UV absorption occurred in early summer. High amounts and diversity of MAAs was also found during red tide of dinoflagellates occurred in the Argentine sea. For instance, during a red tide of *Gymnodinium cf. aureolum* high *in vivo* and *in vitro* absorption in the UV region, with maxima at 334 nm was reported [240] Analysis of samples collected during a red tide of the toxic dinoflagellate *G. catenatum* revealed the net predominance of shinorine, and minor amounts of palythine-serine, porphyra-334, mycosporine-glycine, palythinol and palythene and two unknown UV-absorbing compounds [75]. In *Noctiluca* sp. populations, the most noteworthy feature was the dominance of porphyra-334, which occurred together with several minor MAAs, shinorine, palythine, mycosporine-glycine and palythenic acid [75]. Analysis of samples collected in southern coastal Patagonian waters, in which *P. minimum* was the dominant species, revealed high concentrations of MAAs and the unusual co-dominance of shinorine and usujirene, together with minor amounts of palythene, shinorine methyl ester, porphyra-334 and palythenic acid [228]. Phytoplankton sampled from the English Channel in summer, when high numbers of dinoflagellates, comprising almost exclusively *Gyrodinium aureolum* dominating the phytoplankton biomass, revealed high concentrations of MAAs and a wide diversity of compounds. In particular there were high concentrations of a UV-absorbing compound exhibiting a λ_max_ at 342 nm [111,241]. This compound was also present in cultures of *G. galatheanum* and *G. venificum*. Co-occurring with this compound were high concentrations of mycosporine-glycine, palythenic acid and porphyra-334. In contrast, in phytoplankton extracts from New Zealand coast only mycosporine-glycine and two other unidentified UVR absorbing substances were detected [242].

**Figure 8.**
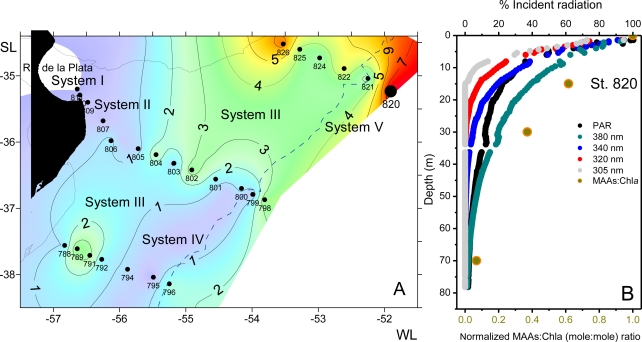
(**A**) Surface distribution of the MAAs/Chl *a* ratio (mole:mole) in the several ecosystems studied [213] across the continental shelf between the Rio de la Plata and the oceanic waters of the Subtropical Convergence. (**B**) Vertical distribution of the MAAs/Chl *a* ratio and PAR and UV light penetration in a location representative of subtropical waters of the Brazil Current [213].

Morrison and Nelson [243] realized a multiyear seasonal study of phytoplankton absorption in the UV range at the Bermuda Atlantic Time-series Study site. Maximum values were observed during summer in surface waters, with peaks between 313 and 335 nm. The seasonal cycle could result either from photoacclimation to change in daily UV exposure or from seasonal succession of phytoplankton with variable MAA concentrations. Interestingly, the summer maximum in UV phytoplankton absorption coincided in this paper, with a predominantly prokaryotic picoplankton community. A seasonal pattern showing a maximum MAA concentration in summer was also reported by Llewellyn and Harbour [241] in phytoplankton extracts from surface waters in the English Channel. In this case, dinoflagellates provided a background of MAAs throughout the year, while peaks of MAAs coincided with blooms of the prymnesiophyte *P. pouchetii* and the diatom *Guinardia striata*. Recently, trends in pigment ratios [214] and MAAs/chlorophyll *a* ratios [213] due to phytoplankton photo-adaptation and photo-acclimation were examined in three sections across the continental shelf between the Rio de la Plata and the oceanic waters of the Subtropical Convergence, during late spring. MAAs were present in all the five different phytoplankton assemblages identified. However, the higher MAAs/(chl *a +* Divinyl (Dv) chl *a*) ratios were recorded at the surface of the oligotrophic subtropical waters where the phytoplankton assemblage showed the dominance of picoplanktonic cyanobacteria *Synechococcus* spp. and *Prochlorococcus* spp. (Figure 8A). This assemblage showed a complex MAA composition including shinorine, palythine, porphyra-334, mycosporine-glycine, palythenic acid, shinorine-methyl ester, and minor amounts of three unidentified compounds [75]. The highest PAR, UVA and UVB transmission were observed at this oligotrophic region, where the euphotic zone reached up to 80 m depth. Consequently, as has been reported for the photo-collector pigments/(chl *a +* Dv chl *a*) and photo-protector pigments/(chl *a +* Dv chl *a*) ratios [214], a sharp transition in the MAAs/chl *a* ratios at depth near the base of the euphotic zone was observed in the water column of this ecosystem (Figure 8B). In contrast, the lowest MAAs/chl *a* ratio were recorded in the inner turbid estuarine waters, where green algae were the dominated group of the phytoplankton assemblage and in the turbulent waters of the shelf break front (Figure 8A).

Steinberg *et al.* [244] examined the production of colored or chromophoric DOM by populations of various planktonic groups from oligotrophic waters and noted that only the cyanobacteria *Trichodesmium* produced CDOM with UV peaks at 325 nm and a shoulder at 360 nm, characteristic of MAAs. Since ocean colour satellite data have captured their large surface blooms in the South Western Tropical Pacific (SWTP) [245], the challenge of optical remote sensing of *Trichodesmium* has stimulated research to determine their optical properties [113] and to quantify *Trichodesmium* from space by convenient algorithms [246,247]. Several bio-optical studies have focused on the UV waveband, in part because harmful algae such as the red-tide dinoflagellates and cyanobacteria mentioned above, produce abundant MAAs and can release them in their environment, making the detection of these compounds interesting for remote detection of harmful blooms. Kahru and Mitchell [248] were the first to examine this possibility in detail, characterizing the remote sensing reflectance for 12 wavelength bands between 340 and 665 nm during a massive bloom of the red-tide dinoflagellate *L. polyedra* off southern California. Recently, a novel algorithm for the early detection of dinoflagellate blooms has been developed using the unique 380 nm spectral band available on the Global Imager (GLI) sensor aboard the Advanced Earth Observing Satellite-II (ADEOS-II) that is not available on other past or present ocean color satellites [249]. The algorithm is based on the approach described earlier [248] and should allow the detection of dinoflagellate blooms at relatively low concentrations.

##### 2.5.3.3. Macroalgae

Many macroalgae produce one or several MAAs [53,54]. Most of the MAA-producing macroalgae belong to Rhodophyceae, followed by Phaeophyceae, and only a few green algae produce MAAs [70,74,75,146,187,191,192,202,203,227,235,250–253]. Generally, red algae might be divided into three different physiological groups related to MAAs synthesis [187]: Members of the first group lack any traces of MAAs, species of the second group contain MAAs in variable concentration dependent of environmental conditions, while algae of the third group always contain a stable and high concentration of MAAs.

It is interesting to note that shinorine and porphyra-334 are by far the most common MAAs reported in macroalgae in species collected from tropical to polar waters [70,74,203,218,254]. However, the MAA compositions of some intertidal red macroalgae, especially from Antarctic waters (*i.e.*, *Palmaria decipiens*, *Iridea chordata*, *Curdiea racovitzae*) were more complex [74,75,191]. For example, in the antarctic red alga *P. decipiens* in addition to shinorine and porphyra-334, palythine, asterina-330, palythinol, palythene, usujirene and the unusual M335/360 were also found [74,75,191]. Even if there is not direct evidence, it is probable that the high diversity of MAAs observed in these species, as discussed for dinoflagellates [10,109], may be consequence of interconversions among primary and secondary MAAs [204]. In the red algae *C. crispus*, MAA synthesis induction by PAR alone follows a pattern, starting with synthesis of shinorine and followed by asterina-330, palythinol and palythine and a decline in shinorine after two days [190,204]. Therefore, asterina-330, palythine and palythinol could be derived products from the shinorine metabolism [190,192,204]. Palythinol might plausibly be formed via carboxylate reduction of the serine residue in shinorine by a presumed reductase enzyme, whereas asterina-330 may be synthesized from shinorine after decarboxylation of the serine subunit [75,163,204]. Krabs *et al.* [192] showed that the synthesis of asterina-330, palythinol and palythene in the red alga *C. crispus* was mainly induced by UVB radiation whereas this radiation had a negative effect on the accumulation of the major MAAs shinorine and palythine. The authors speculate that the enzymes which may catalyse the conversion from palythinol via asterina-330 to palythine, could by inhibited by UVB. Due to the lack of porphyra-334 and usujirene, the pathway for the synthesis of palythene in *C. crispus* remains unclear [192]. However, several species of red algae (e.g., *Palmaria palmata* and *P. decipiens*) including *C. crispus* collected from coastal waters of Maine (USA) contain porphyra-334, usujirene and palythene, but unlike dinoflagellates the *cis* isomer, usujirene appears to be the predominant one [25,108,123]. These diverse responses raise questions about the underlying biochemical trigger for the synthesis induction of palythene and the taxonomic commonality of the process.

In contrast, most marine macroscopic green algae investigated so far lack MAAs [203,235,250]. In an investigation of the occurrence of MAAs in 13 macroalgae Chlorophyceae collected from the intertidal zone of the tropical island Hainan, Karsten *et al.* [255] found a significant concentration of photoprotective compounds, such as mycosporine-glycine and porphyra-334 only in two green algae: *Boodlea composita* and *Caulerpa racemosa*, respectively. However, it was found that the subaerial green macroalgae *Prasiola crispa* sub sp. *antarctica* contain high concentrations of a unique UV-absorbing compound with an absorption maximum at 324 nm, that was characterized as a putative MAAs due to chromatographic properties [250]. Gröniger and Häder [256] confirmed the occurrence of this 324 nm-MAA in the closely related *Prasiola stipitata* from the supralitoral zone of the rocky island Helgoland (North Sea). The ability to produce the unique 324 nm-MAA was exclusively found in the Trebouxiophyceae. It was present in all close relatives of *Prasiola* spp. that were tested as well as in a clade independent of the *Prasiola* allies but was absent in another clade of Trebouxiophyceae that comprised species from terrestrial and freshwater habitats [235].

### 2.6. Biosynthesis and Interconversions of MAAs in Symbiotic Associations

In symbiotic associations it has been inferred that the symbionts are responsible for the synthesis of MAAs [7,19,61,147,162–164,169,170,221]. As has been extensively discussed [7,164,221] differences in MAAs diversity among symbiotic cnidarians may reflect a genotypic difference among zooxanthellae [257] and among hosts. According to Banaszak *et al.* [164] it is highly likely that all clades of *Symbiodinium* can produce MAAs under natural conditions. However the freshly-isolated symbiotic algae, can synthesize a maximum of five MAAs: mycosporine-glycine, porphyra-334, shinorine, palythine and mycosporine-2-glycine [163,164,221], whereas at least 14 different MAAs have been characterized in many coral species [7,61,75,79,103,142,163,164,258–260] with up to 12 MAAs identified in a single coral, *S. pistillata* [75,79,147,163]. Conversely, some *Acrophora* and *Pavona* species contain low amounts and diversity of MAAs, and the primary *Symbiodinium* MAA mycosporine-glycine was the most abundant (up to 95%) compound [7,33,61,84,140,146,162–164,186,261–263]. Mycosporine-glycine was also found in high concentrations in 54 species of symbiotic cnidarians that included hydrozoan corals, anemones, gorgonians and scleractinian corals from the Mexican Caribbean [164]. Therefore, when the host species contains more MAAs than the symbiont, as has been observed in several corals [7,33,75,79,147,163–165,264] the tissue host, and conceivably their associated bacteria, can bio-convert the MAAs produced by the zooxanthellae to yield an array of secondary MAAs [163]. On the contrary, when the host contains fewer MAAs than the symbiont fraction it is possible that some MAAs were not efficiently translocated to the host [164] or were catabolized within the host tissue. Dunlap and Shick [265] for instance, suggested that *Vibrio harveyi* can hydrolyse the hydroxyamino acid substituents of shinorine and porphyra-334, to yield mycosporine-glycine.

In some species such as the corals *Heteroxenia fuscescens* and *Goniastrea retiformis* it would seem that the source of MAAs is not the alga partner of the symbiosis [266,267]. Yakovleva and Baird [267] have suggested that MAA synthesis and conversion of MAAs in planulae of the stony coral *G. retiformis* occurred in the absence of zooxanthellae, suggesting a possible contribution of prokaryotes associated with the animal tissue to these processes. Bacteria are also capable of *de novo* synthesis of MAAs [226] and can provide at least some of the intermediate metabolites, or may be responsible for inter-conversions of MAAs, such as the transformation of primary MAAs to secondary MAAs. However, in various cnidarian species the freshly-isolated zooxanthellae from MAA-containing host, lack MAAs [33,164,165,264], indicating that the MAAs may also be acquired from the diet [19,163–165]. The pathways and the enzymes involved in the bio-transformations of MAAs in symbiotic organisms are still unknown. Shick [163] suggested that in the coral *S. pistillata* mycosporine-methylamine-serine and the structurally related compound mycosporine-*N*-methylamine-threonine could be formed via the aminolysis of serine and threonine from the C1 position of shinorine and porphyra-334, respectively, with these amino acids replacing glycine at C3 in a conjugation addition/elimination reaction. Serine and threonine then would be replaced at C1 by imine formation with ammonium, followed by methylation of the imine. However the main route of shinorine conversion may be the initial decarboxylation of their glycine moiety, followed by demethylation of the mycosporine-*N*-methylamine-serine formed [70,75,79,201], as this pathway has been demonstrated to occur in some cyanobacteria under sulfur deficiency [201]. Similarly, the structurally related, novel compound palythine-threonine found in the coral *P. capitata* and detected in others reef-building coral species including *S. pistillata* [79], may be also formed by the initial decarboxylation of the glycine moiety of porphyra-334 followed by demethylation of the mycosporine-*N*-methylamine-threonine formed [75,79]. However, the biosynthesis of palythine-serine-sulfate in *S. pistillata* remains enigmatic because it is the most concentrated and usually the only MAAs detectable in the coral *S. pistillata*, prior to the UVR MAAs-synthesis induction [163]. Palythine-serine but not palythine-serine-sulfate occurred in natural colonies of the coral *P. capitata* and *Pocillopora eydouxi* [79] what suggests that palythine-serine sulfate may be formed by sulfation of palythine-serine (Figure 6). On the other hand, structural and biochemical considerations also suggest that both palythine-serine and palythine-threonine may be the precursors of the major sulfate esters (palythine-serine-sulfate and palythine-threonine-sulfate) found in some species of scleractinian corals [19,75,79,88,163]. The proposed pathway for conversions of primary MAAs [75,79] can also account for the declining in mycosporine-2-glycine and the concomitant increase, to a similar extent, over the same time course of palythine observed by Shick [163] in the coral *S. pistillata.* For instance, two novel unidentified compounds were detected in extracts of this and other scleractinian corals [75,79,147]. One of them has a maximum absorption at λ = 327 nm—indicative of a chromophore with *N*-methylamine functionality (probably mycosporine-*N*-methylamine-glycine) and may be the product of decarboxylation of one glycine subunits of mycosporine-2-glycine [75,79,147]. The other is probably a “mycosporine sulfate ester” (λ_max_ = 318 nm) [75,79]. Recently, palythine-serine-sulfate and the so-called “mycosporine sulfate ester from *S. pistillata*”, were also detected as minor components, in the non-symbiotic sea anemones *Aulactinia marplatensis* and *Oulactis muscosa* [87], indicating that these compounds are more widely distributed among marine invertebrates than previously found.

In other coral species that also showed a great amount and diversity of MAAs, (*i.e.*, *Montipora aequituberculata*), the secondary products (more that 70% of total MAAs) were palythine, usujirene and palythene [7] which are typically found in dinoflagellates [10,11,23,110,111]. Thus, it is probable that usujirene and palythene in these symbioses, may be formed via dehydration of porphyra-334 (*Z*-palythenic acid) followed by decarboxylation (usujirene) and isomerization of the formed usujirene [10,11,109,123]. A possible explanation for the absence of *Z*-palythenic acid in the coral *M. aequituberculata* could be that divergences exist in the expression of enzymes responsible for the conversion of *Z*-palythenic acid to usujirene. In *A. tamarense* decarboxylation enzymes might be expressed at low levels resulting in a measurable amount of palythenic acid, whereas in the coral *M. aequituberculata* the expression of these enzymes might be high thus converting all palythenic acid to usujirene, so that no palythenic acid is present or it might be present at a concentration below the detection limit. Finally in other coral species that showed a lower diversity of MAAs (*i.e.*, *Montipora verrucosa*), the principal secondary MAAs were palythine, asterina-330 and palythinol [84,142]. Palythinol, might plausibly be formed via carboxylate reduction of the serine residue in shinorine by a presumed reductase enzyme [163], whereas asterina-330 may be synthesized from shinorine after decarboxylation of the serine subunit (Figure 6).

The major MAAs found in symbiotic sea anemones resemble those contained a classic suite of primary compounds seen in other dinoflagellate symbioses (shinorine, porphyra-334 and mycosporine-2-glycine), with the exception of the presence of the unique rare MAA, mycosporine-taurine [14,85–87]. Shick *et al.* [86] showed that the complement of major MAAs in four sea anemones from California in the genus *Anthopleura* (Actiniidae), broadly reflect phylogenetic differences among the anemones rather than taxon of endosymbionts, presence or absence of symbionts or environmental factors. For instance, among *Anthopleura* species the MAAs content and complement of the less closely related species, *A. artemisia* [268] differed from the other species in showing only traces of mycosporine-2-glycine. Similarly, the same complement of major MAAs was observed in the non-symbiotic species *A. marplatensis* and *O. muscosa* (Actiniidae) from Argentine coast. This result together with the absence of mycosporine-2-glycine and high proportions of asterina-330 and mycosporine-glycine, in the non-symbiotic species *Anthothoe chilensis* [86,87] (Sagartiidae) support the phylogenetic hypothesis of Shick *et al.* [86]. For instance, Daly *et al.* [269] showed that family Actiniidae belongs to the *Acontiaria-Boloceroidaria-Mesomyaria* clade, whereas the family Sagartiidae, belongs to the *Endomiyaria* clade. A noteworthy feature is that mycosporine-taurine was found in the highest concentration in all the studied species [14,85–87], a situation that is not altered by controlled diets [86]. Thus, an extra-diet origin of mycosporine-taurine, or perhaps regulated metabolisms of biotransformation of dietary MAAs or their precursors unique to sea anemones should be expected [86,87].

Recent bioinformatics analysis of the genome of the sea anemone *Nematostella vectensis* shows that genes encoding enzymes of the shikimic acid pathway have been transferred to the sea anemone’s genome from both bacterial and eukaryotic (dinoflagellate) donors. Additional genes encoding some enzymes of the shikimic acid pathway are present in a putative, bacterial (Flavobacteriaceae) symbiont of this sea anemone, closely related to those of *Tenacibaculum* sp. MED152 [72]. Moreover, a novel bacterial symbiont species in the genus *Tenacibaculum* (*Tenacibaculum aiptasiae* sp. nov.) was recently isolated from the tropical sea anemone *Aiptasia pulchella* [270]. The incorporation of amino acids to 4-deoxygadusol [82] and to mycosporine-glycine to form primary MAAs is a reversible process [175] as indicated by the bacterial formation of mycosporine-glycine and 4-deoxygadusol from exogenous shinorine and porphyra-334 [265]. Thus, mycosporine-taurine could be synthesized *de novo* [72] or may be produced by incorporation of taurine, the major (∼90%) component of the free amino acid (FAA) pool in symbiotic and asymbiotic anemones [85,271] to dietary or diet derived 4-deoxygadusol and/or by reversible substitution of taurine in the chromophore of the major dietary MAAs, mycosporine-glycine [87]. Taurine is not an exclusive FAA of sea anemones, it occurs in high amounts in all marine invertebrates [272]. Notably, among invertebrates mycosporine-taurine was only detected in sea anemones, indicating that MAAs composition, although related with their free amino acid pool composition [86], was dependent on the expression of non identified specific enzymes encoded into the host genome, involved in the incorporation of taurine to 4-deoxigadusol. Recently, mycosporine-taurine was found in the diatom *P. multiseries* [75], and in the cyanobacteria *Synechocystis* sp. PCC 6803 [34]. Thus, we can expect a wider distribution of mycosporine-taurine as previously found. However, the conclusions that Zhang *et al.* [34] draw from their measurements on the identity of mycosporine-taurine are questionable [73]. Their HPLC method for the analysis of MAAs has neither been used previously nor has it been tested with authentic mycosporine-taurine. The UV absorption spectra that they showed are not at all an indicative of a pure MAA. In addition, this compound has been tentatively identified as mycosporine-taurine based on a detected ion at *m/z* 318 that was asigned to a Na *+* adduct, even though the protonated molecule was not found in the spectrum. On the other hand, chemical characterization of this MAA was incomplete [85] and therefore, their usual identification by comparison with reported UV-absorption maxima, retention times and co-chromatography with a purified substandard derived from *Anthopleura* [75,86,87], should be taken with caution.

In contrast to the large amount of information available for algal-invertebrate symbioses, only a few protist-invertebrate and algal-protist symbioses have been analyzed [46,101,155,170]. The MAAs found in *Maristentor dinoferus*, a benthic ciliate that hosts symbiotic dinoflagellates of the genus *Symbiodinium* of the clade C [273], resemble those contained a classic suite of primary compounds seen in other dinoflagellate symbioses, (mycosporine-2-glycine, shinorine and porphyra-334), with the exception of the presence of the secondary product of porphyra-334 dehydration, palythenic acid [155]. Mycosporine-like amino acids have also been reported in the freshwater ciliate *Stentor amethystinus* Leidy, 1880 that hosts *Chlorella* [274] and, just recently, in several planktonic ciliates bearing *Chlorella* isolated from an oligo-mesotrophic lake [234]. Considering all *Chlorella*-bearing ciliates, Sonntag *et al.* [234] found seven different MAAs (mycosporine-glycine, palythine, asterina-330, shinorine, porphyra-334, usujirene and palythene). Some of these MAAs were also identified in the *Chlorella* isolated from the ciliates, thus providing initial evidence for their symbiotic origin [234].

Non-photosymbiotic ascidians had transparent tunics transmitting both visible and UV light, or pigmented or opaque tunics equally absorb both UV and visible light. However, a prominent absorption peak around 320 nm was exclusively found in the colonial ascidians hosting the algal symbiont *Prochloron* sp. [170,171,275–277]. In the tissues of two colonial didemnid ascidians—*Lissoclinum patella* and *Diplosoma* sp. that contain the symbiotic photo-oxygenic prokaryote *Prochloron* sp., the major MAA (about 94%) was shinorine, and the minor MAA was palythine. Isolated *Prochloron* cells from *L. patella* contained shinorine, which was also dominant in the host tunic [170,171]. However, in *Diplosoma virens*, the composition of MAAs (mycosporine-glycine, palythine, shinorine, and porphyra-334) was different between isolated *Prochloron* cells and colony residue from which *Prochloron* cells were extracted [275]. Recently, the content of MAAs were compared in the two color morphs (dark-gray and brown colonies) of the tropical ascidian *Didemnum molle* (Herdman, 1886), which harbors the photosymbiotic prokaryote *Prochloron* sp. Spectroscopic and chromatographic analyses showed that the *Prochloron* cell density and MAA concentration in the dark-gray colonies were an estimated 1.4 and 2.4 times higher, respectively, than in the brown colonies [277].

Less is known about MAAs metabolism in other cyanobacterial-invertebrate symbioses. Notably, the polyphyletic origin of sponge-associated cyanobacteria indicates that they derived from multiple independent symbiotic events, involving several different cyanobacterial types and/or host sponges [278]. Most of the symbiont sequences were affiliated to a group of *Synechococcus* and *Prochlorococcus* species. However, other symbionts were related to different groups, such as the Oscillatoriales. For instance, *Dysidea herbacea* a shallow marine sponge common in the Great Barrier Reef, that hosts the symbiotic cyanobacterium *Oscillatoria spongelidae* contains an unusual MAA, mycosporine-glutamic acid-glycine as major component [101]. Mycosporine-glycine, usujirene and palythene were the minor components [46,101]. The presence of this unusual MAA in this sponge [101,146,279] may be related to its symbiosis with *O. spongelidae*, a cyanobacterium group for which most MAAs remain undescribed. One exception is the diazotrophic ocean bloom forming cyanobacteria *Trichodesmium* spp. for which was reported to contain high amounts of MAAs. Among those identified, asterina-330 and shinorine were the most abundant, but mycosporine-glycine porphyra-334 and palythene were also found as minor components However, two of the most abundant compounds remain unidentified [113]. Recently, mycosporine-alanine, mycosporine-glutaminol and mycosporine-glutaminol-glucoside previously described in terrestrial fungi, were found in terrestrial Oscillatoriales [76–78]. Interestingly, only oxo-carbonyl mycosporines occur in the fungal-cyanobacterial symbiosis of terrestrial lichens [280]. Globally, the other studied sponges contain a classic suite of primary and secondary compounds. At least six different MAAs (shinorine, porphyra-334, palythine, asterina-330 palythinol and palythenic acid) have been characterized in *Halychondria japonica* from Okinawa Island (Japan) [146], conversely, some Antarctic species (*i.e.*, *Inflatella belli*) only contain palythine [279], the secondary MAAs most wide distributed among sponges.

### 2.7. Uptake, Biotransformation and Accumulation of Maas by Consumer Organisms

The report of genes encoding enzymes for the MAAs biosynthesis pathway in an animal [72], changes the universality of the traditional view that metazoans, as evidenced by their dietary requirement for shikimate-derived aromatic amino acids, don’t have the capacity to synthesize MAAs *de novo* [19]. Moreover, the recent study by Balskus and Walsh [102] showed that the MAA biosyntheses do not involve shikimate pathway intermediates. However the long-standing assumption that consumers acquire, transform and translocate them from their diet has been confirmed experimentally in many metazoans [19,25,167,172,174]. Mammals may be incapable of such. Thus, dietary supplementation with MAAs may be useful in aquacultured species, but MAAs as “dietary sunscreens” may not be an option for mammals, including humans [172].

#### 2.7.1. Selective Uptake

In some organisms, a selective uptake of MAAs from food has been experimentally confirmed [25,126,172]. For example, the sea urchin *Strongylocentrotus droebachiensis* accumulated in the ovaries principally one MAA, shinorine, but not others that were available in high concentration (shinorine, palythine, asterina-330 and usujirene) in their diet, the red macrophyte *C. crispus* [25]. Shinorine is prevalent in the ovaries and eggs of sea urchins from many biomes, including polar [74] boreal [167] and tropical [281]. Conversely, the medaka fish *Oryazias latipes* absorbs and accumulates palythine and asterina-330 but not shinorine from *Mastocarpus stellatus* [172]. The selective uptake of MAAs from food, suggests that in some organisms there are specific transporters for them in the gut [19]. However, almost nothing is known of how dietary MAAs are processed, whether they can be transported and stored, and how they reach their final destinations.

#### 2.7.2. Interconversions of Dietary MAAs

Little information is available on the biotransformation of MAAs through pelagic organisms, particularly the transfer of MAAs from phototrophic phytoplankton to primary consumers [242,282,283]. In the heterotrophic and phagotrophic dinoflagellate *Noctiluca scintillans* several common MAAs (mycosporine-glycine, shinorine, porphyra-334, palythenic acid and palythine) have been reported. The most noteworthy feature is the presence of both (*Z/E*) palythenic acid isomers [75], yet *E*-palythenic acid isomer was never observed in marine phytoplankton. Both compounds were first isolated in the phagotrophic dinoflagellate *Noctiluca* sp. (Okaichi and Tokomura in [92]) and then in the ascidia *Halocynthia roretzii* [92]. Moreover, their presence in the Antarctic krill *Euphausia superba* and other primary consumers [146] indicates that *Z/E* isomerization takes place in many organisms during the process of MAAs assimilation. Whitehead *et al.* [152] reported that the number of MAAs (mycosporine-glycine, shinorine, porphyra-334, palythenic acid and palythine) was greater in the herbivorous pteropod (*L. helicina*) *versus* the natural phytoplankton assemblage (shinorine and porphyra-334), and that an apparent increase in total MAAs concentration enriched in palythine was produced during transference to the predatory pteropod *Clione antarctica*. In contrast, Newman *et al.* [174] observed that both the type and concentration of MAAs in the Antarctic krill *E. superba* reflect the MAA composition and concentration of their experimental phytoplankton diet, *P. antarctica*. These results would imply that concentrations of MAAs in naturally occurring populations of krill would depend on, and vary with, concentrations of MAAs in phytoplankton assemblages that it is feeding on. However natural populations of the New Zealand krill *Nyctiphanes australis* were found to contain five identified common MAAs, mycosporine-glycine, shinorine, porphyra-334, palythine, and palythinol and several unknown UV-absorbing compounds with little indication of a seasonal cycle that could be correlated with either phytoplankton MAAs (consisting almost entirely in mycosporine-glycine) concentration or ambient UV irradiances [242]. An explanation is that although MAAs cannot be retained indefinitely, they can remain in body tissues for long periods with minimal dietary supplementation [174]. In contrast, Hylander *et al.* [282] showed that copepods upregulated their MAA content when UV threat was increasing (*i.e.*, if MAAs were abundant in food), and in field data this accumulation only occurred at high levels of predation threat. If MAAs were scarce, copepods instead compensated with higher carotenoid accumulation. However, when there was a high predation threat this carotenoid compensatory effect was disadvantageous, and low concentrations of both MAAs and carotenoids at high UV-threat resulted in lower reproduction. On the other hand, García *et al.* [283] found that MAA accumulation in freshwater copepods may occur even if the copepods are cultured on a MAA-free diet and that the bacteriostatic antibiotic, chloramphenicol, inhibits the bioaccumulation of MAAs. The authors suggest that the source of MAAs in these copepods may be prokaryotic organisms in close association with the animals.

MAAs have been found concentrated in the epidermis, ocular tissue, eggs and reproductive tissues of animals [19,118,265,284], but in the New Zealand krill *N. australis* MAAs concentrations throughout the body (legs, eyes, antennae, muscle) were similar, with the exception of the caparace that had a much lower concentration [242]. The lower concentration in the caparace is unexpected given it is the surface that other protective compounds, such as carotenoids have been found to be concentrated [285]. According to Riemer *et al.* [242], the lower caparace MAAs concentrations could be due to removal of compounds during moulting and to the time required for MAAs to be incorporated into new caparace tissue. However, several studies showed that the amounts and composition of MAAs accumulated in different tissues of metazoans are particular and differ from that in their diets [19]. Asterina-330 is usually a major MAA component in the epidermis of sea cucumbers, yet this MAA is absent from their diet of benthic microalgae and filamentous cyanobacteria, which contain mostly shinorine, mycosporine-2 glycine, and porphyra-334. In the coral reef sea cucumber *Thelenota ananas*, the MAAs complement in the foregut corresponds to that in dietary microflora with the lumen of the digestive tract showing decreasing concentrations of algal MAAs and increased concentrations of mycosporine-glycine. Asterina-330 is rarely present in the gut content but reaches high concentration in the intestinal tissue and especially in the epidermis [19]. These authors demonstrated that bacteria from the enteric fluids of *T. ananas* were responsible for MAAs interconversions. The epidermal tissues of the sea cucumber *Holothuria atra* also contained several MAAs, many in significant amounts [47]. Mycosporine-glycine, palythine, asterina-330, and shinorine were the major MAAs, while mycosporine-2-glycine, porphyra-334 and palythinol were minor components. Mycosporine-glycine was the major MAA in the epidermal tissues, in the gut contents and the diet, and the only MAA present in the ripe ovaries and the viscera. Mycosporine-glycine and palythine were the only quantifiable MAAs present in the gut contents and sediment [47]. It is therefore reasonable to predict that the aerobic bacterial flora in the gut may also be the provider of at least some MAAs that could be accumulated in the epidermal tissues (*i.e.*, asterina-330). On the other hand, mycosporine-glycine could be selectively accumulated and stored in the viscera and later “transferred” to the ovaries. Gadusol, which co-occurs with MAAs, and in certain instances with trace amounts of 6-deoxy-gadusol in unfertilized and fertilized eggs and developing larvae of some marine invertebrates and vertebrates, was absent from *H. atra*. However, 6-deoxygadusol, the proposed biosynthetic precursor of MAAs, was present, but only in the ovaries [47]. In a recent study quantitative survey of MAAs in intertidal egg masses from temperate rocky shores from Australia (46 species of molluscs, two species of polychaetes and one species of fish), Przeslawski *et al.* [284] showed that MAAs occurred in relatively high concentration in intertidal molluscs egg masses when compared to adult mollusc and other common intertidal organisms. Adult diet, phylogeny and viability affected MAA composition. Herbivores had higher levels of certain MAAs than carnivores, but MAA composition varied according to the taxonomic group [284].

The lenses of many shallow water fish also absorb short wave radiation and the pigments in these lenses have been identified as MAAs. These pigments appear more widespread and have been isolated from lenses of fish from a variety of habitats. Pigments extracted from 52 species have been identified as palythine, asterina-330, palythinol and palythene. In the majority of species asterina-330 was the most abundant compound, but the amounts and composition of MAAs varied among species. Gadusol, which is structurally related to the MAAs, is present in the fish ovaries that contain ripe eggs just before spawning, and is a minor component in the fish lenses [118]. Three MAAs were also found in the lens and cornea of the cuttlefish *S. officinalis*. Palythinol were found in the lens and cornea, whereas palythene were limited to the lens and shinorine was identified only in cornea [119].

MAAs were also found in the external mucus of fish [173,286–288]. Their presence in fish mucus appears to be a general characteristic of many reef fishes, with 90% of the coral reef fish species surveyed from Hawaii, the Great Barrier Reef, and Johnston Atoll showing UV absorbance in their mucus [288]. Recently, Eckes *et al.* [173] showed that the amounts and composition of MAAs (asterina-330, palythene and mycosporine-*N*-methylamine serine) in coral reef fish mucus not only varied among species but also within species sampled in different locations, indicating that diet and other associated factors may regulate the type and quantity of MAAs found in the mucus of reef fishes. MAAs in coral mucus is the first-line barrier developed by corals against solar radiation. For example, in the solitary coral *Fungia fungites*, the presence of UV-absorbing material in coral mucus was showed to be positively correlated with the solar flux [289]. Up to nine MAAs compounds were present in coral mucus of seven species of corals from French Polynesia: Palythine, mycosporine-glycine, mycosporine-2-glycine, shinorine pophyra-334, palythinol, palythine-serine, mycosporine-methylamine-serine, and mycosporine-methyl-amine-threonine [142]. The correspondence between the relative concentration of nine MAAs in mucus and in the whole coral, suggests that for most species there is not a preferential active excretion of one particular MAAs [142].

## 3. Conclusions

Taxonomically diverse marine and terrestrial organisms have evolved the capacity to synthesize, accumulate and metabolize a variety of mycosporine-like amino acids as part of an overall strategy to diminish the direct and indirect damaging effects of environmental ultraviolet radiation. Recent phylogenetic analysis revealed that genes that might be involved in the first steps of the biosynthesis of MAAs were transferred from cyanobacteria to dinoflagellates and finally to metazoan by horizontal gene transfer, indicating the evolutionary significance of these compounds during the course of evolution. However the long-standing assumption that consumers acquire, transform and translocate them from their diet has been confirmed experimentally in many metazoans.

Details of the pathway and the enzymes involved in the biosynthesis, transference, accumulation and transformations of more than 20 fully characterized MAAs and of the recently discovered compounds, are still unknown and further studied are required. Nevertheless, there is biochemical evidences to assume that the high diversity of MAAs present in marine organisms is principally derived from the synthesis and transformation of the so-called “primary MAAs”.

The identification and quantitative determination of each MAA in marine organisms is of crucial importance to scientific progress in the field. The inherent optical properties of MAAs, makes it very difficult to distinguish these compounds based on absorption spectra only. However, the major difficulty has been the lack of commercially available standards and therefore, identification and quantification are most usually based on co-elution with secondary standards and/or comparison with published UV spectral data and HPLC retention times. Such characterization of these compounds should be treated with caution. Mass spectrometry coupled to chromatography, calibrated with certified references, can provides high sensitivity and selectivity, making these assays superior to the ones depending on conventional detection technologies. In applying LC-MSn instead of single stage mass spectrometry, an even higher degree of selectivity can be achieved, making a mass spectrometric distinction of isomeric compounds possible.

Several physicochemical and physiological studies demonstrated that the sunscreen role is the most important function that MAAs and other mycosporines play in nature. However, a full understanding of their function should also take the less known aspects into account. In addition to the ecological significance of MAAs as sun-screening substances, these compounds have potential applications in cosmetics and toiletries as a UV protectors and activators of cell proliferation with therapeutic properties that may be exploited in a large amount of commercial applications.

## Figures and Tables

**Table 1.** Methodologies used for characterization of the most common MAAs.

**MAAs**	**Chemical Assay**	**m.p. Determination**	**Elemental Analysis**	**UV-Vis Spectroscopy**	**Optical Activity**	**IR Spectroscopy**	**MS Spectroscopy**	**NMR Spectroscopy**	**X Ray Diffraction**	**References**
asterina-330	• Methylation • H/D Interchange	No	No	Yes ɛ determined	No	No	EI-MS (high resolution) ESI-MS (high resolution) ESI-MSn	^1^H-NMR	No	[17,97–99]
dehydroxylusujirene	No	No	No	Yes	No	No	ESI-MS/MS	No	No	[34]
euhalothece 362	• Amino acid composition after acid hydrolysis	No	No	Yes	No	No	ESI-MSn (high resolution)	No	No	[77]
mycosporine-2-glycine	• Amino acid composition after acid hydrolysis	No	No	Yes	No	No	ESI-MSn (high resolution)	^1^H-NMR ^13^C-NMR	No	[77,85,100]
mycosporine-glutamic acid-glycine	• Amino acid composition after alcaline hydrolysis	Yes	No	Yes ɛ determined	No	No	SI-MS	^1^H-NMR ^13^C-NMR	No	[101]
mycosporine-glycine	• Amino acid composition after neutral or alkaline hydrolysis • Methylation	Yes after derivatization	No	Yes ɛ determined	Yes after derivatization	No	ESI-MS (high resolution)	^1^H-NMR ^13^C-NMR	No	[67,101,102]
mycosporine-glycine-valine	• Amino acid composition after alkaline hydrolysis	No	No	No	No	No	No	No	No	[74]
mycosporine-methylamine-serine	No	No	No	Yes ɛ determined	No	No	ESI-MSn	^1^H-NMR	No	[84]
mycosporine-methylamine-threonine	• Amino acid composition after hydrolysis (not detailed)	No	No	Yes ɛ determined	No	No	ESI-MS (high resolution)	^1^H-NMR ^13^C-NMR	No	[103]
mycosporine-taurine	• Amino acid composition after hydrolysis (not detailed)	No	No	Yes	No	No	ESI-MS	No	No	[85]
palythene	• MAA residue after acid hydrolysis. • Catalytic hydrogenation	Yes	Yes	Yes ɛ determined	Yes	Yes	ESI-MSn	^1^H-NMR ^13^C-NMR	Yes	[96,99,104]
palythine	• Amino acid composition after alcaline hydrolysis • Interchange H/D	Yes	Yes	Yes ɛ determined	Yes	Yes	ESI-MS (high resolution) ESI-MSn	^1^H-NMR ^13^C-NMR	Yes	[95,98,99, 104,105]
palythine-serine	No	No	No	Yes. ɛ determined	No	No	ESI-MSn	^1^H-NMR	No	[84]
palythine-serine sulfate	No	No	No	No	No	No	ESI-MS (high resolution)	^1^H-NMR ^13^C-NMR	No	[88]
palythine-threonine sulfate	No	No	No	No	No	No	ESI-MS (high resolution)	^1^H-NMR ^13^C-NMR	No	[88]
palythine-threonine	• Amino acid composition after alkaline hydrolysis • Interchange H/D	No	No	Yes	No	No	ESI-MS (high resolution) ESI-MSn	No	No	[79]
palythinol	• Amino acid composition after alkaline hydrolysis • Methylation • Interchange H/D	Yes	Yes	Yes. ɛ determined	Yes	Yes	ESI-FTICR-MSn (high resolution) ESI-MSn	^1^H-NMR ^13^C-NMR	No	[98,99,104,106]
porphyra-334	• Amino acid composition after alkaline hydrolysis • Methylation.	No	Yes	Yes. ɛ determined	Yes	Yes	ESI-MSn	^1^H-NMR ^13^C-NMR	No	[90,91,99]
shinorine	• Amino acid composition after acid hydrolysis • Interchange H/D	Yes	Yes	Yes. ɛ determined	Yes	Yes	ESI-MS (high resolution) FD-MS ESI-MSn	^1^H-NMR ^13^C-NMR	No	[98,99,107]
usujirene	• Amino acid composition after alkaline hydrolysis • MAA residue after acid hydrolysis • Catalytic hydrogenation	Yes	No	Yes	Yes	Yes	FD-MS (high resolution) FAB-MS (high resolution)	^1^H-NMR ^13^C-NMR	No	[36,108]
*Z*-palythenic acid	• Amino acid composition after hydrolysis (not detailed)	Yes	No	Yes ɛ determined	Yes	No	FD-MS ESI-MSn	Yes	No	[92,99]
